# IMAGE: INTEGRATE-Mediated *Agrobacterium* Genome Engineering

**DOI:** 10.3389/fmicb.2025.1676008

**Published:** 2025-11-06

**Authors:** Ephraim Aliu, Liang-Chun Chen, Keunsub Lee, Kan Wang

**Affiliations:** 1 Department of Agronomy, Iowa State University, Ames, IA, United States; 2 Crop Bioengineering Center, Iowa State University, Ames, IA, United States; 3 Interdepartmental Plant Biology Major, Iowa State University, Ames, IA, United States

**Keywords:** *Agrobacterium rhizogenes*, *Agrobacterium* spp., *Agrobacterium tumefaciens*, Cre-*lox*, CRISPR integrase, disarming, large DNA fragment deletion, targeted DNA insertion

## Abstract

The ability to precisely engineer *Agrobacterium* strains is crucial for advancing their utility in plant biotechnology. We recently implemented the CRISPR RNA-guided transposase system, INTEGRATE, as an efficient tool for genetic modification in *Agrobacterium*. Despite its promise, the practical application of INTEGRATE in *Agrobacterium* strain engineering remains underexplored. Here, we present a standardized and optimized workflow that enables researchers to harness INTEGRATE for targeted genome modifications. By addressing common challenges, such as crRNA design, transformation efficiency, and vector eviction, this protocol expands the genetic toolkit available for *Agrobacterium*, facilitating both functional genomics and strain development for plant transformation. As a demonstration, we domesticated *Agrobacterium rhizogenes* K599 strain by deleting the 15-kb T-DNA region from its root-inducing plasmid pRi2659 and inactivating a thymidylate synthase gene to render the strain auxotrophic for thymidine. The protocol provides detailed guidance for each step, including target site selection, crRNA spacer cloning, *Agrobacterium* transformation, screening for targeted insertion and Cre/*loxP*-mediated deletion, and vector removal. This resource will empower new users to perform efficient and reproducible genome engineering in *Agrobacterium* using the INTEGRATE system, paving the way for broader adoption and innovation in plant biotechnology.

## 1 Introduction

*Agrobacterium tumefaciens* is a fundamental tool in plant biotechnology, widely utilized for genetic transformation thanks to its natural ability to transfer DNA (T-DNA) into plant genomes. This soil-borne, gram-negative bacterium exists as a free-living organism but can adopt a pathogenic lifestyle upon acquiring a tumor-inducing (Ti) plasmid (Goodner et al., 2001; Wood et al., 2001; Gelvin, 2003). In nature, pathogenic *Agrobacterium* recognizes wounded plant tissues, where phenolic compounds from damaged cells trigger the expression of virulence (*vir*) genes on the Ti plasmid (Braun, 1952). The *vir* genes facilitate T-DNA processing and delivery into host cells via the Type IV Secretion System (T4SS). Once inside the host nucleus, the T-DNA integrates into the plant genome, driving phytohormone overproduction and tumor-like crown gall formation (Gelvin, 2003).

Disarmed and engineered *A. tumefaciens* strains are now widely used for plant genetic engineering, enabling advancements in crop improvement, functional genomics, and synthetic biology (Gelvin, 2003; Aliu et al., 2022, 2024; Kang et al., 2022, 2024). Efforts to optimize *Agrobacterium* strains using homologous recombination (HR), transposon mutagenesis, and CRISPR technologies continue to expand their utility and host range. As a result, *Agrobacterium* remains central to innovation in plant science.

HR-based genetic modification has been instrumental in engineering *Agrobacterium* strains for research and industrial use. For instance, the AGL1 strain was generated via double-crossover HR to replace the *recA* gene with a deactivated *recA* allele generated by ampicillin resistance gene (*bla*) insertion (Lazo et al., 1991). This mutation reduces unwanted spontaneous recombination in cosmid-based binary vectors and improves overall plasmid stability, especially for constructs with repetitive sequences (Lazo et al., 1991). However, *recA* knockout limits further genomic modifications via HR (Lazo et al., 1991; Goralogia et al., 2025).

Auxotrophic *Agrobacterium* strains have also been developed to improve plant transformation efficiency and biosafety. Disruption of essential biosynthetic pathways renders these strains dependent on supplemented media, enabling tighter growth control and reducing bacterial overgrowth and carryover. These strains lower the need for high-dose antibiotics and address biosafety concerns linked to recombinant DNA technologies. As they cannot survive outside laboratory conditions, their use in delivering genome-editing tools like CRISPR/Cas helps mitigate the risk of environmental release. Notable examples include HR-based knockouts of *thyA*, *metA*, and *IVLC* genes, which confer thymidine, methionine, and valine-isoleucine auxotrophy, respectively (Ranch et al., 2012; Prías-Blanco et al., 2022; Aliu et al., 2024; Yang et al., 2025).

Transposon (Tn) mutagenesis has played a pivotal role in *Agrobacterium* modification. For example, the widely used LBA4404 strain was derived from LBA4213 through Tn904 insertion into the Ti plasmid of the wild-type Ach5 strain (Klapwijk Van Breukelen et al., 1980; Ooms et al., 1982). This approach also facilitated the identification of numerous genes essential for T-DNA transfer (Dale et al., 1993; Huang et al., 1990; Kang et al., 1992; Stachel and Nester, 1986; Goralogia et al., 2025). However, both HR and transposon mutagenesis have limitations: HR is labor-intensive and requires sequence homology, while transposon insertions are random, making targeted modifications and downstream strain optimization more challenging (Aliu et al., 2024; Tamzil et al., 2021).

The CRISPR/Cas-based approaches have transformed bacterial genome engineering, but most systems rely on nuclease-induced double-strand breaks (DSBs), which can cause toxicity and unintended mutations (Komor et al., 2016; Rodrigues et al., 2021; Goralogia et al., 2025). To address these issues, base editors have emerged as an alternative, enabling precise single-nucleotide transitions without introducing DSBs (Komor et al., 2016). In *Agrobacterium*, Rodrigues et al. (2021) adapted a cytidine base editor based on the Target-AID system from *E. coli* (Banno et al., 2018), consisting of a catalytically inactive Cas9 fused to the cytidine deaminase CDA1. Expression was placed under the *virB* promoter (PvirB), inducible via the VirG regulator, enabling conditional genomic site-specific C-to-T conversions in the presence of plant signal molecules. This system has been used to generate *recA* knockouts and auxotrophic mutants, demonstrating its potential for precision strain engineering (Rodrigues et al., 2021; Pennetti et al., 2025). However, leaky expression from PvirB can result in background mutations even without induction (Rodrigues et al., 2021). Additionally, off-target and bystander effects remain a concern, with up to 60 unintended base edits observed in coding regions (Rodrigues et al., 2021; Goralogia et al., 2025). Moreover, editing efficiency also varies by chromatin accessibility and sequence context (Arbab et al., 2020), highlighting the need for more efficient, precise tools to enhance *Agrobacterium* strain performance for plant transformation.

Recent advances in CRISPR-associated transposase (CAST) systems have expanded the toolkit for programmable DNA integration, offering new avenues for genome engineering beyond traditional nuclease-based approaches (Klompe et al., 2019; Strecker et al., 2019; Vo et al., 2021). Among these, Type V CASTs rely on a single catalytically inactive Cas12k effector. A representative example is the Type V-K system from *Scytonema hofmanni*, which utilizes Cas12k in conjunction with a trans-activating CRISPR RNA (tracrRNA) (Strecker et al., 2019). Unlike canonical Tn7 transposons, this system lacks the TnsA subunit, resulting in cointegrate formation during transposition (Tou et al., 2023). Additionally, the Type V-K systems exhibit off-targeting and a preference for integration near tRNA genes (Saito et al., 2021), which may influence target site selection in host genomes. In contrast, Type I CASTs offer a more precise and efficient cut-and-paste transposition mechanism (Klompe et al., 2019; Vo et al., 2021; Gelsinger et al., 2024). The INsertion of Transposable Elements by Guide RNA–Assisted TargEting (INTEGRATE) system exemplifies this class, combining a nuclease-deficient type I-F CRISPR/Cas system with the Tn6677 transposon from *Vibrio cholerae* (Klompe et al., 2019; Vo et al., 2021; Gelsinger et al., 2024). This system employs a multi-subunit Cascade complex alongside TnsA, TnsB, and TnsC, enabling site-specific DNA integration at 48–50 bp downstream of a CRISPR RNA (crRNA)-guided target site without introducing double-strand breaks (Klompe et al., 2019). This system has shown high precision and efficiency in bacterial genome modifications, including *E. coli*, *Klebsiella oxytoca*, and *Pseudomonas putida* (Vo et al., 2021).

To extend this approach to *Agrobacterium,* we optimized a single-plasmid INTEGRATE system containing the full Cas–transposition operon, single or multiple crRNAs, and a donor mini-Tn flanked by engineered transposon ends carrying customizable cargo (Aliu et al., 2022). We also included a *sacB* cassette as a counterselection marker to allow for post engineering plasmid curing (Aliu et al., 2022). Unlike traditional HR, Tn, or conventional CRISPR-base editing approaches, INTEGRATE enables high-fidelity, marker-free genome modifications across various *Agrobacterium* strains. Using this system, we achieved targeted knockouts of *recA* and *thyA* in EHA101, EHA105, and the previously recalcitrant AGL1 strain (Aliu et al., 2024), with high efficiency. Moreover, we demonstrated multiplexed insertions and large-fragment deletions by combining INTEGRATE with site-specific recombinases, generating disarmed wild-type variants (Aliu et al., 2022). Despite its utility, INTEGRATE’s broader application is limited by strict protospacer adjacent motif (PAM) requirements, target-site constraints, transposon orientation biases, potential cargo remobilization, and variable efficiency in multiplexed targeting and deletions.

Here, we present a comprehensive protocol for INTEGRATE-mediated genome engineering in *Agrobacterium*. The protocol includes a case study in which the system is used to disarm the T-DNA and engineer a thymidine auxotrophic *Agrobacterium rhizogenes* K599 strain. This platform supports efficient, precise, and stable genetic modifications, advancing strain development for plant transformation, functional genomics, and biotechnology applications.

## 2 Materials and equipment

### 2.1 Agarose gel electrophoresis

- 100 bp DNA ladder (NEB, cat. no. N3231L)
- Agarose, low melting temperature (Research Products International, LOT 147134-15,562)
- Gel casting set (Fisherbrand, cat. no. 14-955-209)
- Gel loading dye, orange 6 × (Thermo Scientific, cat. no. FERR0631)
- Gel loading dye, purple 6 × (NEB, cat. no. B7024A)
- GeneRuler 1 kb plus DNA ladder (Thermo Scientific, cat. no. FERSM1332)
- RedSafe nucleic acid staining solution (Bulldog-Bio, cat. no. 21141)
- TAE buffer, 50 × (Thermo Scientific, cat. no. B49)
- TopVision Agarose (Thermo Scientific, cat. no. R0492)

### 2.2 Antibiotics

- Carbenicillin disodium (Gold Biotechnology, cat. no. C-103-100)
- Kanamycin Monosulfate (Fisher Scientific, cat. no. BP906-5)
- Spectinomycin Dihydrochloride Pentahydrate (Gold Biotechnology, cat. no. S-140-25)

### 2.3 Bacteria growth media

- CMG Buffer (See Supplementary materials)
- Luria-Bertani (LB) (Fisher Scientific, cat. no. BP1426-2)
- Super Optimal Broth (SOB) (See Supplementary materials)
- Super Optimal Broth with Catabolite Repression (SOC) (ThermoFisher, cat. no. 15544034)
- Terrific Broth (TB) (See Supplementary materials)
- Yeast Extract Peptone (YEP) (See Supplementary materials)

### 2.4 Bioinformatic tools and useful links

- Benchling (www.benchling.com)
- Biomath molar online calculator (www.promega.com/resources/tools/biomath/)
- ClustalW (https://www.ebi.ac.uk/jdispatcher/msa/clustalo)
- Restriction Enzyme digestion (https://nebcloner.neb.com/#!/)
- Geneious (www.geneious.com)
- Gibson Assembly (www.biocat.com/bc/files/Gibson_Guide_V2_101417_web_version_8.5_x_11_FINAL.pdf)
- National Centre for Biotechnology Information (NCBI) BLAST (https://blast.ncbi.nlm.nih.gov/Blast.cgi)
- NEBio ligation calculator (https://nebiocalculator.neb.com/)
- NEB melting temperature (Tm) calculator (https://tmcalculator.neb.com/)
- Primer3 (https://primer3.ut.ee/)
- Restriction Mapper (https://www.restrictionmapper.org/)
- SnapGene (https://www.snapgene.com/)

### 2.5 Biological materials

- *Agrobacterium* electrocompetent cells
  - Intact genomics
  - Gold Biotechnology
- *E. coli* chemical super-competent cells
  - Subcloning Efficiency™ DH5α (ThermoFisher, cat. no. 18265017)
  - High-Efficiency NEB® 5-alpha (NEB, cat. no. C2987H, C2987I)
  - ig® 5 alpha chemically competent cells (Intact genomics).
- Plasmid list (Supplementary Figure S1)

### 2.6 Cloning

- *Bsa*I–HFv2 restriction enzyme and rCutSmart™ buffer (NEB, cat. no. R3733S/L)
- *Xho*I restriction enzyme and rCutSmart™ buffer (NEB, cat. no. R0146S/L/M)
- *Pst*I–HF restriction enzyme and rCutSmart™ buffer (NEB, cat. no. R3140S/T/L/M)
- NEBuilder® HiFi DNA Assembly Master Mix (NEB, cat. no. E2621S/L/X)
- rCutSmart buffer (10X) (NEB, cat. no. B6004S)
- T4 DNA Ligase (NEB, cat. no. M0202S/T/L/M) and 10X ligase buffer (NEB, cat. no. B0202S)
- T4 Polynucleotide Kinase (PNK; NEB, cat.no. M0201S/L)
- Thermosensitive Alkaline Phosphatase (TSAP) (Thermo Scientific, EF0654)

### 2.7 DNA extraction and polymerase chain reaction (PCR)

- 0.2 mL PCR tubes with caps (Greiner bio-one, cat. no. 608201)
- 96 well PCR plate (Fisherbrand, cat. no. 14230232)
- Cap strips for PCR plate (Thermo Scientific, cat. no. AB-0451)
- Mini Centrifuge (Fisher Scientific, cat. no. 05-090-100)
- Oligonucleotide/primers (Integrated DNA Technologies or preferred vendor)
- Q5 High-Fidelity 2X Master Mix (NEB, cat. no. M0492S/L)
- Taq 2 × Master Mix for genotyping only (NEB, cat. no. M0270L)
- Thermocycler (Analytikjena, cat. no. Biometra TAdvanced) or preferred vendor.
- Sterile nuclease-free water such as UltraPure™ DNase/RNase-Free Distilled Water (Invitrogen, cat. no. 10977015)

### 2.8 Other equipment/instruments/reagents

- 0.22 μm syringe filter (Fisherbrand, cat. no. 09-720-004).
- 1.5- and 2-mL Eppendorf tubes (Fisher Scientific or preferred vendor)
- Dimethyl sulfoxide (DMSO) (MilliPORE SiGMa, cat. no. 20-139)
- Electrophoresis system, Enduro™ Gel XL (Labnet, cat no. E0160)
- Ethanol, Absolute (Fisher Scientific, cat. no. BP28184)
- Gel Documentation System (Axygen, GDBL-1000)
- Heat block (Thermolyne, Type 17,600)
- Laminar flow hood (NuAire, NU-340)
- Liquid nitrogen (Chemistry store, Iowa State University or preferred vendor)
- Nanodrop Spectrophotometer (Marshall Scientific, ND-1000)
- Pipette tips (Fisherbrand SureOne, cat. no. 02-707-438/02-707-403/02-707-417)
- Spectrophotometer (Thermo Scientific, GENESYS 10S UV–VIS)
- Tabletop centrifuge (Eppendorf, cat. no. cat. no. 5420 or 5424R)
- Ultra-low temperature freezer (So-Low, U80-28)
- Vortex mixer (Fisher Scientific, cat. no. 02215365)
- Water bath (Stellar Scientific, SL-SWB-27)

### 2.9 Stock solution and media preparation

Stock solution and media preparation are provided in the Supplementary materials.

## 3 Methods

**Figure 1:**
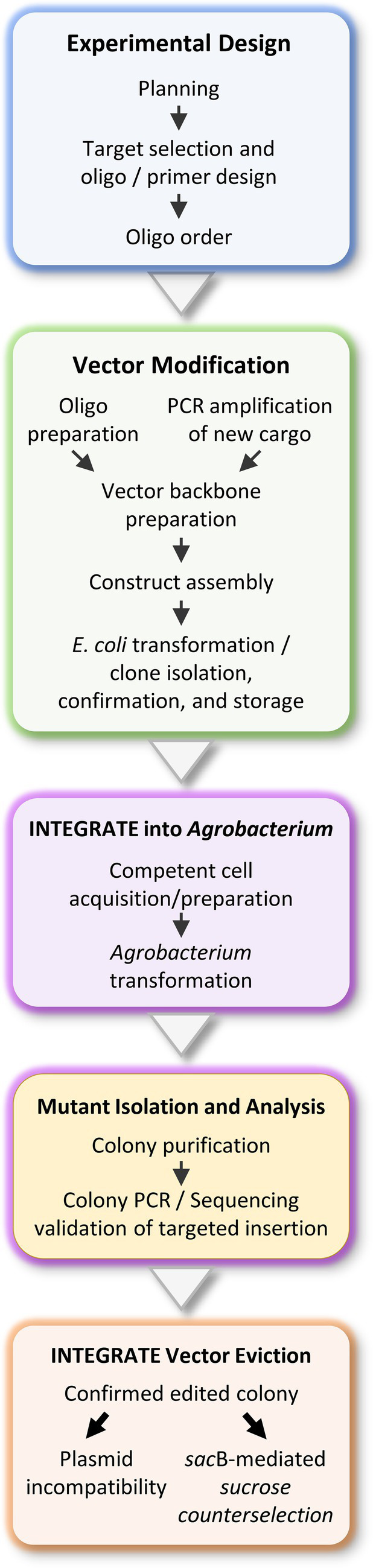
Summary of the CRISPR RNA-guided integrase-mediated genome engineering workflow for *Agrobacterium* strain modifications.

The INTEGRATE protocol provides a stepwise guide for efficient genome engineering in *Agrobacterium*, organized into five sections (Figure 1): (A) Experimental design, (B) Vector modifications, (C) *Agrobacterium* transformation, (D) Mutant isolation and analysis, and (E) INTEGRATE vector eviction. Successful execution requires core molecular biology skills, including Polymerase Chain Reaction (PCR), agarose gel electrophoresis, DNA purification, bacterial transformation, culture, and aseptic techniques. While not mandatory, familiarity with molar ratios is helpful; online tools are referenced as needed. Users must be proficient in primer design and gene-editing software for accurate cloning and target validation. Access to nucleotide sequences of the target gene is essential for guide RNA selection and edit verification. While the protocol is broadly applicable, strain-specific optimization is critical for maximizing success. A strong understanding of the targeted *Agrobacterium* strain’s biology will greatly enhance transformation efficiency and overall protocol performance.

### 3.1 Experimental design

#### 3.1.1 Target site selection and guide RNA (crRNA) design

**Figure 2:**
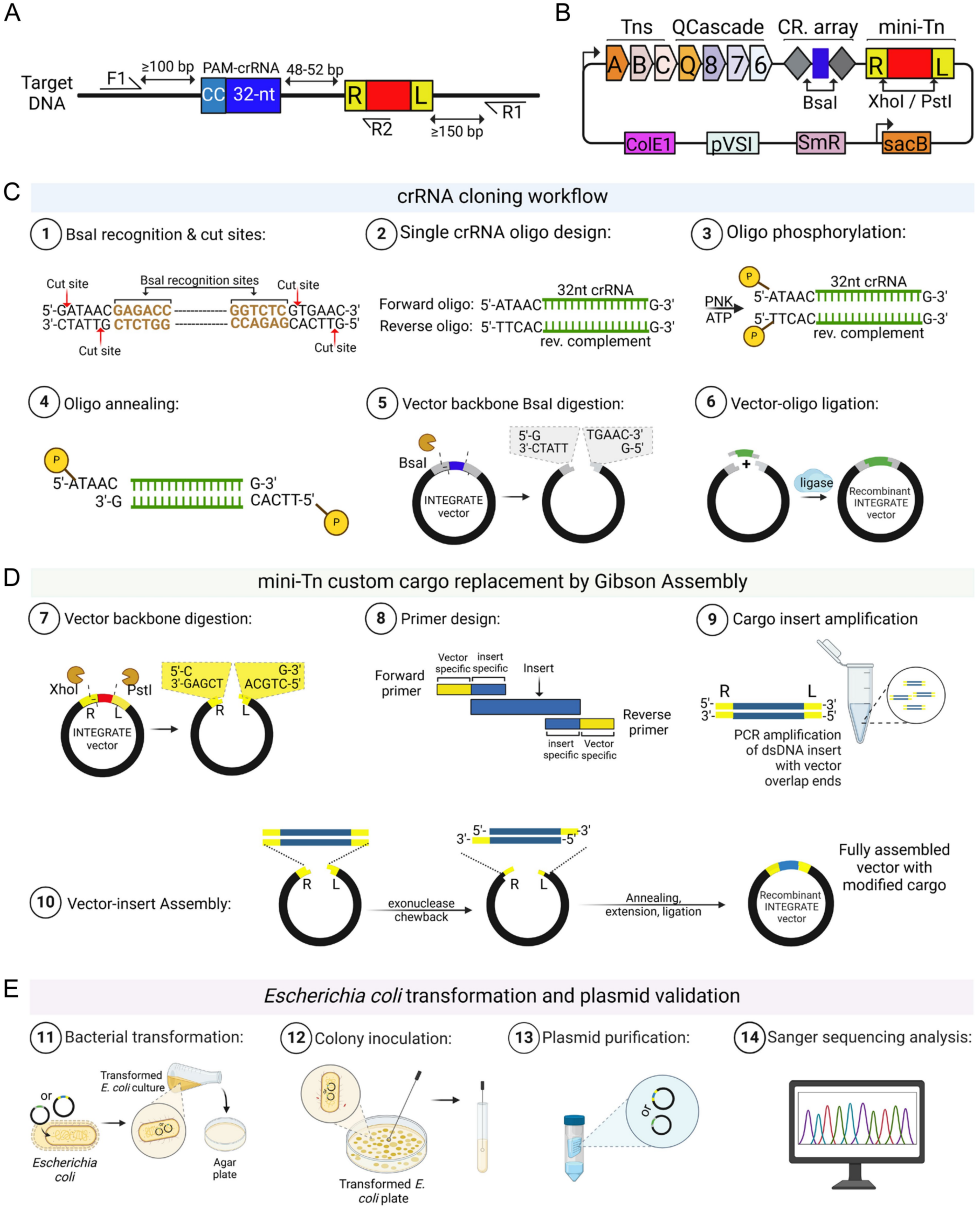
INTEGRATE vector construction. **(A)** The *Vibrio cholerae* INTEGRATE system recognizes a dinucleotide -CC- as the PAM (protospacer adjacent motif) sequence. The following 32 bases are selected as the crRNA (CRISPR RNA) targeting guide for *Agrobacterium* genome engineering. Integration takes place 48–52 base pairs downstream of the target site. F1 is the forward primer, which is recommended to be designed at least 100 base pairs upstream of the crRNA recognition sequence. R1 is the reverse primer, recommended to be designed at least 150 base pairs downstream of the anticipated transposon insertion site. The R2 reverse primer can be used in combination with either F1 or R1 to determine the orientation of the integrated transposon. **(B)** For custom Agrobacterium engineering, the INTEGRATE vector can be modified at the *Bsa*I site for crRNA cloning into the CRISPR array, and at the *Xho*I/*Pst*I sites for cargo modification. The vector illustrated is a schematic of the pEA186 vector, which has been deposited on Addgene (ID: 187874). This vector contains Spectinomycin and *sacB* selection markers, along with a pVS1 backbone. **(C)** A summary of the *Bsa*I-mediated ligation cloning process for the designed crRNA oligonucleotides is shown. **(D)** Cargo replacement can be performed using Gibson Assembly, and a summary of this procedure is also provided. **(E)** After successfully cloning the crRNA and custom cargo, the final step is to transform or propagate your cloned vector in *E. coli*, followed by validation through Sanger sequencing as described in the main text. Schematic representations were created using Biorender.

For *Agrobacterium* genome engineering, clearly defining the experimental objective is crucial, as it directly influences target site selection, crRNA design, integration specificity, and overall editing efficiency (Figure 1). A key prerequisite for precise INTEGRATE-mediated insertion is the presence of a protospacer adjacent motif (PAM) immediate upstream of the target sequence (Figure 2A). The Type I-F INTEGRATE system uses a TniQ-Cascade complex and transposase proteins (TnsABC) to specify the integration site and mediate cargo DNA transposition (Figure 2B; Klompe et al., 2019; Vo et al., 2021). This system recognizes a 5’-CC-3’ PAM, and the 32-nt protospacer sequence immediately downstream of this PAM is used as a crRNA guide (Figure 2A). In *Agrobacterium*, Tn insertions predominantly occur at 48–52 bp downstream of the protospacer (Aliu et al., 2022). Therefore, to ensure precise DNA insertion, a 32-nt protospacer sequence must be located 48–52 bp upstream from the desired insertion site (Figure 2A).

While INTEGRATE demonstrates high specificity – achieving near-perfect accuracy in *Agrobacterium tumefaciens* C58 and its derivatives (Aliu et al., 2022, 2024), *Agrobacterium rhizogenes* K599 (this study), and LBA4404 (Aliu et al., 2024) – off-target insertions remain possible, especially in non-*Agrobacterium* strains where low-frequency off-target events have been reported (Vo et al., 2021). Off-targeting is more likely in genomic regions with high sequence similarity to the crRNA target, particularly those containing conserved PAM sequences. Studies in *E. coli* suggest that the INTEGRATE QCascade complex tolerates mismatches in the crRNA guide’s seed region (positions 1–8) and PAM-distal region (positions 25–32) with minimal impact on specificity. However, mismatches in the central region significantly impair targeting accuracy and efficiency (Gelsinger et al., 2024). Therefore, selecting target sites in *Agrobacterium* should follow these mismatch tolerance principles and be thoroughly validated for specificity. For sequenced *Agrobacterium* strains, online bioinformatics tools such as NCBI BLAST (BLAST: Basic Local Alignment Search Tool, https://blast.ncbi.nlm.nih.gov/Blast.cgi) and Primer-BLAST (Primer designing tool, https://www.ncbi.nlm.nih.gov/tools/primer-blast/) can be used to predict potential off-target sites. For unsequenced strains, whole-genome sequencing using platforms such as Plasmidsaurus™ or other commercial sequencing services is recommended prior to target selection and crRNA design. This ensures accurate off-target analysis and enhances the precision of INTEGRATE-mediated modifications.

#### 3.1.2 Choosing the right tool for the task – vector selection

To facilitate *Agrobacterium* strain engineering, we have developed a set of optimized INTEGRATE plasmids, including vectors designed for gene insertion, insertional mutagenesis, and the deletion of large DNA fragments (Supplementary Figure S1; Aliu et al., 2022).

1. Vector for gene insertion or insertional mutagenesis: pEA186 (Addgene #187874; Figure 2B; Supplementary Figure S1). If the goal is to insert a gene cassette into bacterial chromosomes or plasmids, or to generate an insertional mutagenesis on the genome, pEA186 is a suitable vector choice. It is based on a pVS1 origin of replication (ORI) backbone and is engineered to facilitate site-specific genomic integration of the INTEGRATE mini-transposon (mini-Tn) cargo in *Agrobacterium*. It includes a *mCherry* reporter gene driven by the constitutive J23107 promoter (Noh et al., 2017), allowing real-time visualization of the plasmid presence. Additionally, it carries the *sacB* gene (Gay et al., 1983; Steinmetz et al., 1983), enabling sucrose-induced plasmid eviction post-genome engineering. The vector confers spectinomycin (Sp) resistance and features a customizable CRISPR array, which can be modified via dual *Bsa*I sites (Figure 2B; Supplementary Figure S1). The *mCherry* cargo is also interchangeable, as it can be replaced using *Pst*I and *Xho*I restriction sites (Figure 2B; Supplementary Figure S1). Following genomic integration, pEA186 can be efficiently removed from *Agrobacterium* through *sacB*-mediated counterselection on 5% sucrose medium (see Section 3.5).
2. Vectors for large DNA fragment deletion: pKL2310 (Addgene #187875) and pKL2315 (Addgene #187876). If the purpose is to generate large deletions on the chromosomes, the pKL2310/pKL2315 system is recommended for optimal performance. pKL2310 is similar to pEA186 but specifically designed for targeted genomic deletions. Instead of the *mCherry* reporter, it carries a 34-bp *loxP* sequence as its cargo and lacks the *sacB* gene. Consequently, pKL2310 cannot be evicted via *sacB*-mediated counterselection; instead, it is displaced through plasmid incompatibility with pKL2315. After successful dual *loxP* insertions at the desired genomic locus, the second step involves introducing pKL2315, a Cre recombinase-expression vector, to facilitate pKL2310 eviction and catalyze precise genomic deletion via Cre-*loxP* recombination. Built on a pVS1 backbone, pKL2315 carries a kanamycin (Km) resistance gene and includes the *sacB* gene, allowing its subsequent removal following successful deletion.

### 3.2 INTEGRATE vector modification

This section involves cloning the designed 32-bp crRNA guide sequence into the selected INTEGRATE vector described in Section 3.1.2. If desired, the genetic payload on the mini-Tn can also be modified. The guide sequence (i.e., spacer) is cloned between the two CRISPR repeats using dual *Bsa*I restriction sites (Figures 2B,C-1). Users may choose to clone either the cargo (Figure 2D) or crRNA spacer first (Figure 2C), but must avoid introducing new restriction sites, particularly *Bsa*I, which could disrupt downstream steps. If the cargo introduces additional *Bsa*I sites, the crRNA spacer should be cloned first. Tools like RestrictionMapper v3 (https://www.restrictionmapper.org/) or Snapgene’s enzyme noncutter function can help identify problematic sites early in the design process.

#### 3.2.1 Design and prepare crRNAs for single or multiplexed targets

For single crRNA cloning, oligonucleotides must be designed with precise overlaps for seamless insertion into the CRISPR array of pEA186 or pKL2310 (Figure 2C-1-2). The forward oligonucleotide (5′ to 3′) should include:

- A 5-nt extra sequence (5’-ATAAC-3′) at the 5′ end, matching the deleted CRISPR repeat sequence with compatible 4-nt overhang for the vector ends generated by *Bsa*I digestion, followed by,
- A 32-nt spacer sequence, and
- A 1-nt “G” at the 3′ end to compensate for the loss of the first “G” from the right CRISPR repeat after *Bsa*I digestion.

Similarly, the reverse oligonucleotide (5′ to 3′) should include:

- A 5-nt extra sequence (5’-TTCAC-3′) at the 5′ end,
- The reverse complementary of the 32-nt spacer sequence, and
- A 1-nt “G” overlap at the 3′ end to compensate for the loss of the first “G”-nt from the left CRISPR repeat after *Bsa*I digestion.

Example 1: Insertional mutagenesis of *thyA* gene in *A. rhizogenes* K599 to generate thymidine auxotrophic strain.

*thyA* target sequence (PAM-Protospacer): 5’-CCGGCATGTCATGGAAACCGGCTCCGACCGCGGA-3′.

thyA-Forward: 5’-**ATAAC**GGCATGTCATGGAAACCGGCTCCGACCGCGGA**G**-3′.

thyA-Reverse: 5’-**TTCAC**TCCGCGGTCGGAGCCGGTTTCCATGACATGCC**G**-3’.

Part of the CRISPR repeat sequences are shown in bold.

For multiplexed genomic targeting, crRNA arrays can be designed for dual, triple, tetra, or higher-order integrations. As shown in Supplementary Figure S2, oligonucleotides should be designed such that each 32-nt crRNA spacer sequence is flanked by CRISPR repeats. Additionally, unique 4-nt overhangs should be included to facilitate efficient directional ligation of multiple oligo duplexes. Accordingly, we provide adaptable oligonucleotide designs in Supplementary Table S1 for single (ID4, 5) dual- (ID6-9), triple- (ID10-15), and quadruple- (ID16-23) multiplexed CRISPR arrays.

Example 2: Dual *loxP* insertion to disarm *A. rhizogenes* K599.

LB target sequence (PAM-Protospacer): 5’-CCGAAACGTGCTCCCTCATGAAAAGGTCGCGAAT-3’.

LB-Forward: 5’-**ATAAC**GAAACGTGCTCCCTCATGAAAAGGTCGCGAAT**GTGAACTGCCG**-3’.

LB-Reverse: 5’-**TACTCGGCAGTTCAC**ATTCGCGACCTTTTCATGAGGGAGCACGTTTC**G**-3’.

RB target sequence (PAM-Protospacer): 5’-CCGCGCTTGCCTGATTTGAGAGGTTGTCTCTGCA-3’.

RB-Forward: 5’-**AGTAGGTAGCTGATAAC**GCGCTTGCCTGATTTGAGAGGTTGTCTCTGCA-3’.

RB-Reverse: 5’-**TTCAC**TGCAGAGACAACCTCTCAAATCAGGCAAGCGC**GTTATCAGCTACC**-3’.

Part of the CRISPR repeat sequences are shown in bold.

**Figure 3:**
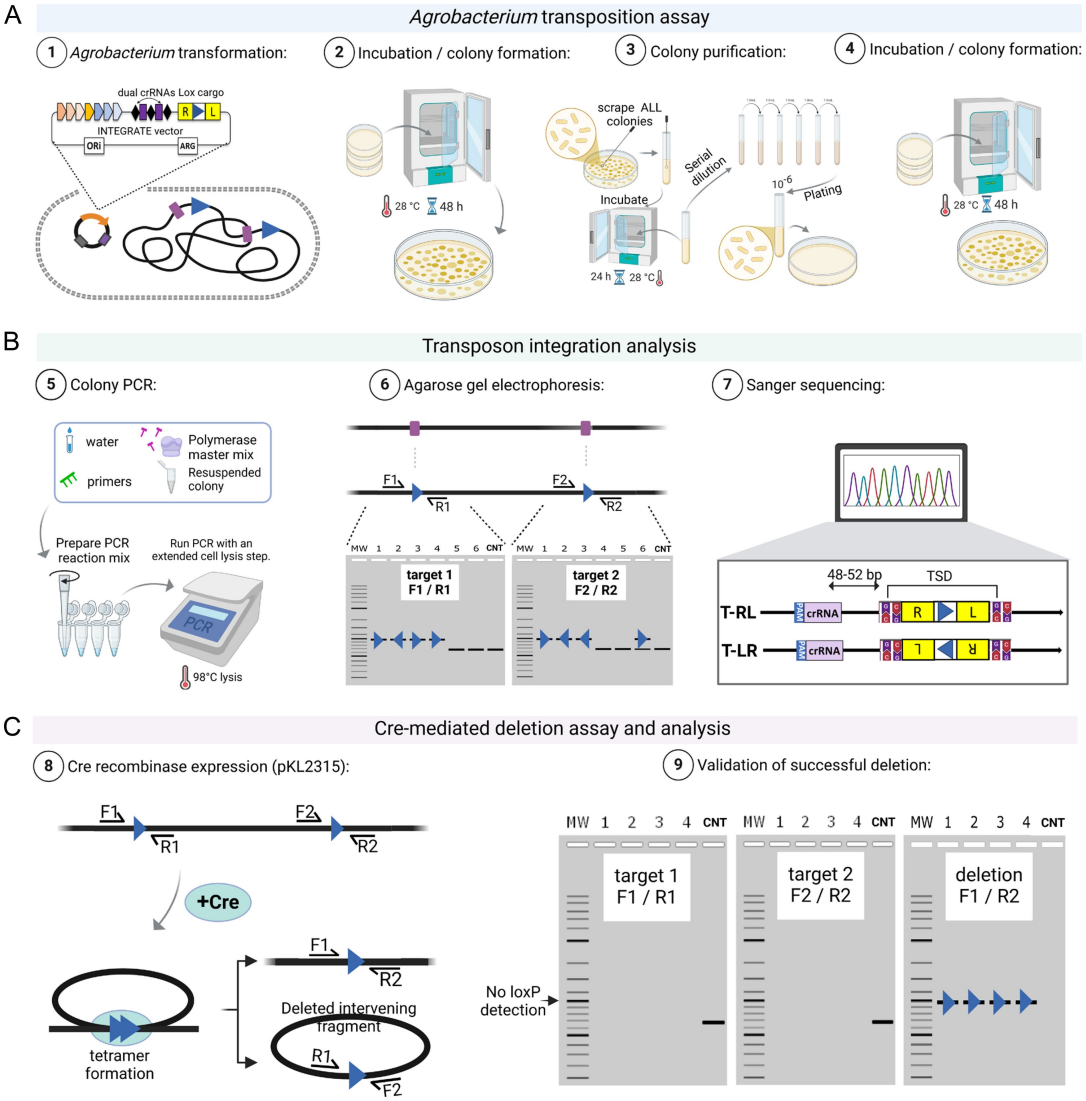
INTEGRATE-mediated *Agrobacterium* genome engineering. **(A)** In Step 1, an INTEGRATE vector containing a *loxP* mini-transposon (Tn) cargo, along with dual CRISPR RNAs (crRNAs) complementary to a specific genomic target site, is introduced into *Agrobacterium*. In Steps 2 to 4, the resulting colonies undergo a purification process (Aliu et al., 2022) to generate a new set of colonies. **(B)** These colonies are then analyzed in Step 5 through colony PCR to identify integration events. In Steps 6 and 7, colonies exhibiting dual *loxP* insertions in the same orientation are selected for Cre-mediated deletion in panel **(C)**. Steps 8 and 9 show cre-mediated targeted deletion. After the recombination event, the reverse (target 1, R1) and forward (target 2, F2) primer binding sites will no longer be present, meaning that no DNA band will be visible after PCR amplification. However, PCR using forward (target 1, F1) and reverse (target 2, R2) primers will confirm successful deletion, with a residual *loxP* site still detectable. A hypothetical agarose gel electrophoresis result is shown for demonstration. CNT represents the negative control. Schematic representations were created using Biorender.

To enhance ligation efficiency, oligonucleotides should be phosphorylated (Figure 2C-3) before further downstream processing, either during synthesis or enzymatically using T4 PNK.

**Figure 4:**
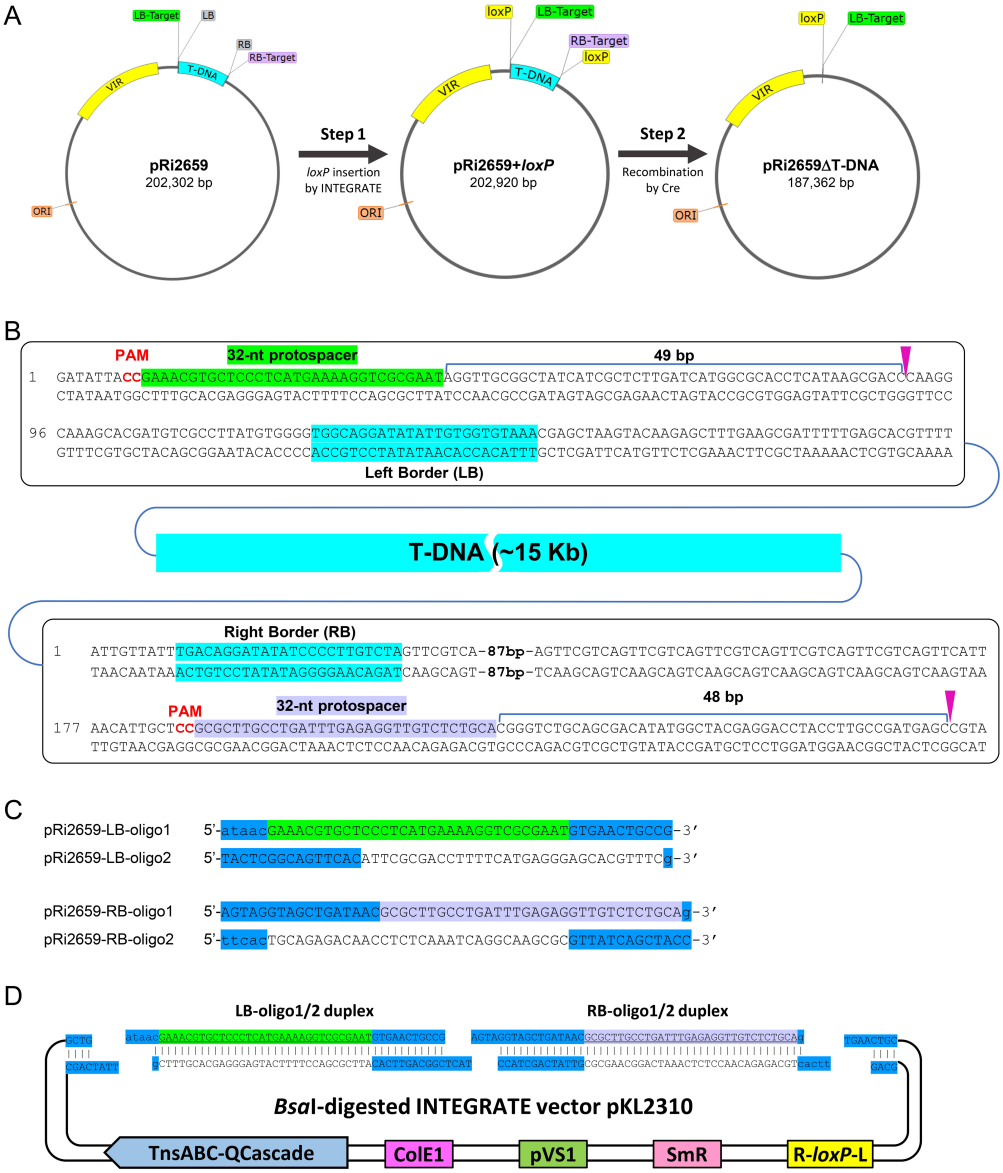
INTEGRATE/Cre-*loxP*-mediated disarming of *Agrobacterium rhizogenes* K599. **(A)** Schematic illustration of the T-DNA region deletion in pRi2659. **(B)** Guide RNA design and targeted insertion sites in LB (top) and RB (bottom) flanking regions. **(C)** Oligonucleotides designed for LB (green) and RB (light purple) target sites. CRISPR repeat sequences are highlighted in blue. **(D)** Spacer cloning into INTEGRATE vector via ligation.

1. Synthesize custom-designed forward and reverse oligonucleotides using preferred DNA synthesis service providers.
2. Dissolve oligonucleotides in sterile nuclease-free water to a final concentration of 100 μM. To achieve this, add a volume of water that is 10 × the oligo’s nanomole amount. For example, if an oligo is supplied as 22.8 nmol, add 228 μL of water to dissolve it.
*Oligos are typically shipped in dry or lyophilized form, which can result in partial adherence to the tube cap. To avoid loss of material, briefly centrifuge the tubes before opening to ensure the entire contents are collected at the bottom.*
3. Phosphorylate oligos using T4 PNK if oligonucleotides are ordered without 5′-phosphate modifications. Set up a 50 μL reaction in a 0.2 mL PCR tube for phosphorylation as follows:
  - Sterile nuclease-free water: 34 μL
  - 10x T4 DNA ligase buffer (with ATP): 5 μL
  - Forward oligo (100 μM): 5 μL
  - Reverse oligo (100 μM): 5 μL
  - T4 PNK (10 units/μL): 1 μL
  - Total: 50 μL.
  Mix by pipetting or vortexing and collect reaction mixture by a brief centrifugation, and then incubate at 37 °C for 30 min. Inactivate the PNK enzyme at 65 °C for 20 min. The phosphorylated oligos can then be stored at −20 °C or used immediately for annealing.
4. Anneal phosphorylated oligos (Figure 2C-4) by heating the reaction mixture to 95 °C for 5 min using a thermocycler or heat block to denature the oligos, and then gradually cool down the mixture to 25 °C at a rate of 0.1 °C per second to promote proper annealing. Once the temperature reaches 25 °C, place the annealed oligoduplex on ice for immediate use or store it at −20 °C for future applications.

#### 3.2.2 Preparation of pEA186 or pKL2310 entry vectors for crRNA cloning

**Figure 5:**
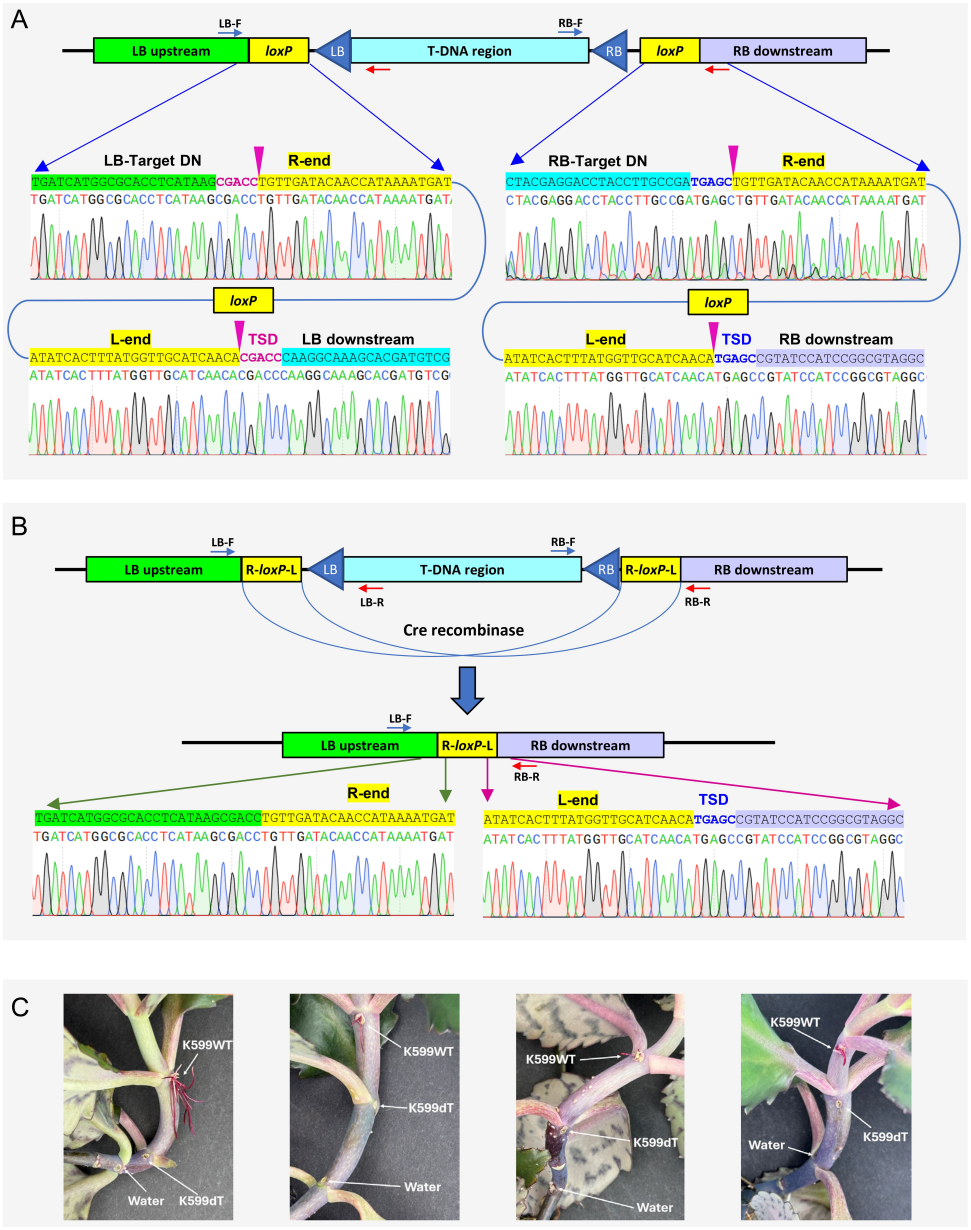
Targeted deletion of the T-DNA region in *Agrobacterium rhizogenes* K599 and mutant characterization. **(A)** Targeted insertion of *loxP* into LB (left) and RB (right) target sites. Chromatograms reveal exact insertion locations (indicated by pink arrow). TSD, target site duplication. **(B)** Deletion of T-DNA region via Cre recombinase mediated recombination. Chromatograms confirm the precise recombination and removal of the 15-kb T-DNA region. **(C)** Characterization of disarmed K599ΔT-DNA strain (K599dT) by Kalanchoe hairy root induction assay. Purple hairy roots were induced by the K599WT (wild-type) strain but not by the disarmed strain (K599dT) or water control (Water). All three strains carry pLC112K plasmid, which includes betalain biosynthesis marker *RUBY*.

**Figure 6:**
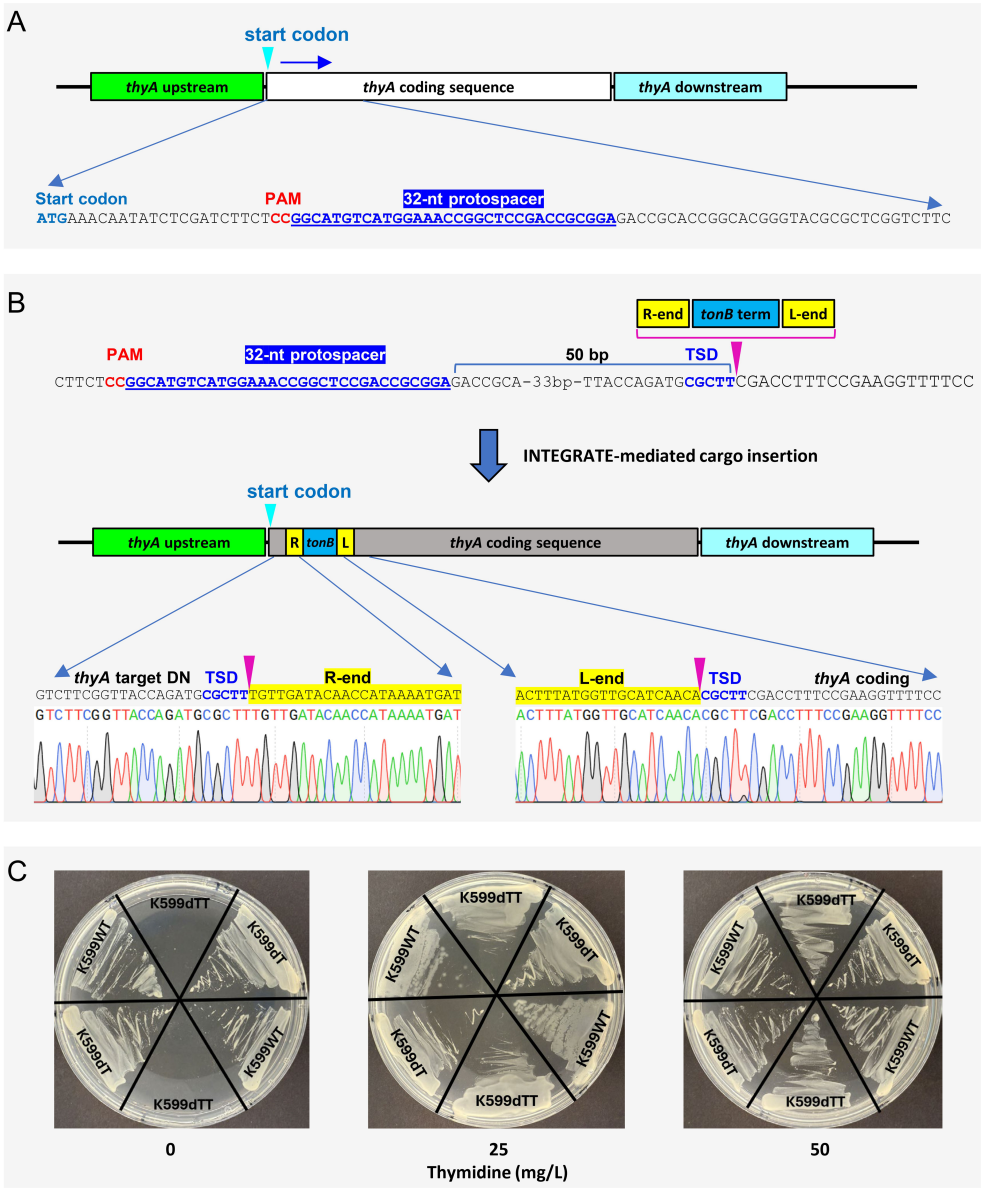
Generation of thymidine auxotrophic K599ΔT-DNA strain via INTEGRATE-mediated insertional mutagenesis of *thyA*. **(A)** Guide RNA design for the thymidylate synthase gene *thyA*. **(B)** Targeted insertion of the mini transposon into the *thyA* coding region. Chromatograms reveal the exact insertion location (indicated by the pink arrow). TSD, target site duplication. **(C)** Thymidine-dependent growth of *thyA* mutant (K599dTT). K599WT, wild-type K599; K599dT, disarmed K599ΔT-DNA; K599dTT, *thyA* knockout mutant of disarmed K599 strain.

1. Digest pEA186 or pKL2310 vector DNA (Supplementary Figure S1) with restriction enzyme *Bsa*I in a microcentrifuge tube as follows (Figure 2C-5):
  - 10x rCutSmart® Buffer: 5 μL
  - Vector DNA (up to 5 μg): x μL
  - *Bsa*I-HFv2 (20 units/μL): 1 μL
  - Sterile nuclease-free water: 44 - x μL
  - Total: 50 μL.
  Gently mix the reaction by pipetting up and down and microfuge briefly. Incubate the tube for 3 h at 37 °C. For optimal digestion, incubate the reaction overnight or increase the enzyme concentrations if needed.
2. Run the digestion mixture on 1% (w/v) agarose gel and perform electrophoresis to separate the DNA fragments. Visualize the gel under UV or Blue lights and excise the large DNA fragment in the gel (~ 17.8 kb for pEA186 or ~ 15 kb for pKL2310) using a sterile blade. Transfer the gel pieces to a 1.5-mL microcentrifuge tube.
  Bsa*I digestion generates two DNA fragments (one large and one small) for pEA186 or pKL2310. The small 38 bp fragments may not be visible by 1% agarose gel electrophoresis.*
3. Purify the *Bsa*I digested vector DNA using NEB/Qiagen Gel Extraction kit or other preferred commercial kits per the manufacturer’s protocol (see Materials). Measure recovered DNA concentration using a NanoDrop spectrophotometer.
*Always verify the purity of gel-extracted DNA by measuring the absorbance ratio at 260 nm and 280 nm. A 260/280 ratio of 1.8–2.0 is considered pure for DNA. A lower ratio suggests contamination with proteins, phenol, or other substances. A secondary check is the 260/230 ratio, which should be ~2.0–2.2. Lower values indicate contamination with salts or organic solvents, which can interfere with ligation efficiency. Refer to the gel extraction kit manual for troubleshooting.*
4. Set up a 10 μL reaction to ligate single crRNA oligoduplex (see Section B1) and *Bsa*I digested vector DNA (see Section 3.2.2 Step 1–3) as follows (Figure 2C-6):
  - 10x T4 DNA ligase buffer (with ATP): 1 μL
  - crRNA oligoduplex (100 pmol/μL): 1 μL
  - Vector DNA (0.01 pmol*): x μL
  - T4 PNK (10 units/μL): 1 μL
  - Sterile nuclease-free water: 7 – x μL
  - Total: 10 μL.
  *Amount of vector DNA for 0.01 pmol: 117 ng for pEA186/*Bsa*I and 100 ng for pKL2310/*Bsa*I.
5. Set up a 10 μL reaction to ligate two crRNA oligoduplexes as follows:
  - 10x T4 DNA ligase buffer (with ATP): 1 μL
  - crRNA1 oligoduplex (100 pmol/μL): 1 μL
  - crRNA2 oligoduplex (100 pmol/μL): 1 μL
  - Vector DNA (0.01 pmol*): x μL
  - T4 PNK (10 units/μL): 1 μL
  - Sterile nuclease-free water: 6 – x μL
  - Total: 10 μL.
  *Amount of vector DNA for 0.01 pmol: 117 ng for pEA186/*Bsa*I and 100 ng for pKL2310/*Bsa*I.
6. Incubate the ligation reaction at room temperature (22 °C) for 30 min after mixing gently by vortexing or pipetting. After ligation, the reaction product is ready for *Escherichia coli* transformation or can be stored at −20 °C for future use.
  *Inefficient ligation can cause recombination between the two Vch CRISPR repeats, leading to the loss of one repeat instead of proper crRNA insertion. Optimizing ligation conditions improves spacer insertion efficiency to over 90%. For multiplexing* (*>2 oligos*)*, extend ligation to 45–60 min or add more T4 DNA ligase* (e.g.*, 2 μL*).
7. Transform *E. coli* (DH5α) using 1 to 5 μL of the ligation mixture and 50 μL of thawed competent cells in a 1.5-mL microcentrifuge tube. Gently flick to mix and incubate on ice for 30 min. Heat shock at 42 °C for 30 to 45 s, then immediately chill on ice for 2 min.
  Add 250 μL of Super Optimal Broth (SOB) or LB medium to the tube. Incubate at 37 °C shaker incubator (200 rpm) for 1 to 2 h. After outgrowth, centrifuge the transformed cells at 10,000–15,000 rpm for 30 s in a benchtop centrifuge. Discard 200 μL of the supernatant and resuspend the pellet in the remaining 100 μL. Plate the entire volume onto LB agar containing the appropriate antibiotic (100 mg/L spectinomycin for pEA186 and pKL2310). Incubate overnight at 37 °C.
8. Pick at least four colonies and inoculate each into 5 mL of LB medium with the appropriate antibiotics in 50 mL conical tubes. Incubate at 37 °C with shaking at 200 rpm for 16–18 h. Extract plasmid DNA using a miniprep kit following the manufacturer’s instructions.
9. Verify the crRNA spacer cloning by Sanger sequencing using the primer pSL1765-seq-F2 (Supplementary Table S1) or custom-designed primers specific to the cloning region. Analyze sequencing results using the software tools recommended in the Materials section or other preferred bioinformatics tools.
*When feasible, sequence the entire plasmid to confirm correct insert incorporation without unwanted mutations or rearrangements, ensuring reliable performance in downstream applications.*

#### 3.2.3 Modifying the INTEGRATE mini-Tn payload/cargo

To insert custom genetic cargo into the INTEGRATE mini-Tn, the pEA186 or pKL2310 vector can be modified using *Xho*I and *Pst*I sites via standard cloning methods such as Gibson assembly (Gibson et al., 2009) or directional cloning (Figure 2D-7-10). [*While natural CRISPR-associated Tn can exceed 100 kb* (Peters et al., 2017; Rybarski et al., 2021; Gelsinger et al., 2024)*, the upper mobilization limit in Agrobacterium is unknown. We have successfully inserted Tn cargos ranging from ~0.3 to 10 kb, with optimal transposition efficiency observed between 0.3 kb and 3 kb. Larger cargos tend to have lower efficiency. The following section provides a step-by-step protocol for replacing the mini-Tn cargo in pEA186 or pKL2310.*]

For Gibson assembly, design primers with vector-specific overlap sequences (20–30 bp for inserts up to 5 kb; longer for larger inserts). Thus, we recommend adding the following extra bases to the 5′ end of a forward primer (5’-GTAAGTTTACGACATTTTCCTCGAG-3′) and reverse primer (5′- GAGTATTTCAGCAAAACTACTGCAG-3′), respectively. If the insert DNA is amplified from genomic DNA, we recommend a two-step PCR to minimize nonspecific amplifications. The first round PCR is performed using the designed target-specific primers and the second round PCR is done using the primers with extra bases. Although generally less efficient, directional cloning remains a reliable method for Tn cargo replacement. To enable directional ligation, add *Xho*I (5’-CTCGAG-3′) and *Pst*I (5’-CTGCAG-3′) recognition sites and the cut-off sequences of the Tn ends to the 5′ ends of forward (5’-CCGCTCGAG-3′) and reverse (5’-TACTGCAG-3′) primers, respectively. After PCR, digest the amplified fragments with both enzymes to generate compatible overhangs. Since restriction enzymes have reduced activity near the end of DNA fragments, we included extra bases (four for *Xho*I and two for *Pst*I, respectively) to the 5′ end of the recognition sites. The optimal number of extra bases varies by enzyme and can be determined using experimentally validated recommendations, such as those provided by New England Biolabs (NEB) (see Cleavage Close to the End of DNA Fragments, https://www.neb.com/en-us/tools-and-resources/usage-guidelines/cleavage-close-to-the-end-of-dna-fragments). Additionally, we provide customizable sequences (Supplementary Table S1; ID24, 25) that can be adapted for forward and reverse primer design.

##### 3.2.3.1 Insert DNA preparation

1. Design primers using web-based tools such as Primer3 or NCBI Primer-BLAST. Aim for a GC content of 40–60% and avoid secondary structures or primer-dimers (to check for self or cross dimer formation, see Multiple Primer Analyzer, Thermo Fisher Scientific, US). The melting temperature (Tm) of forward and reverse primers should be within 5 °C of each other to ensure balanced binding. Significant Tm differences can reduce amplification efficiency and yield. Use BLAST to confirm primer specificity and avoid off-target binding. Add proper overlapping sequences to the 5′ end of forward and reverse primers (see Supplementary Table S1).
2. Dissolve primers in sterile nuclease-free water to prepare 100 μM stocks and dilute 10 μL with 90 μL of sterile nuclease-free water (10 μM working stock) and mix thoroughly by vortexing or pipetting.
3. Set up a 25 μL PCR reaction on ice in a 0.2 mL PCR tube.
  - 2x Q5 master mix: 12.5 μL
  - Forward primer (10 μM): 1.25 μL
  - Reverse primer (10 μM): 1.25 μL
  - Template DNA*: 1 μL
  - Sterile nuclease-free water: 9 μL
  - Total: 25 μL.
  *Template DNA: If using plasmid DNA as template, choose one with a different selection marker than the target vector or remove the plasmid DNA after PCR using restriction enzyme Dpn*I, which specifically cleaves methylated DNA. To minimize background in downstream cloning, use ≤5 ng of plasmid DNA as PCR template. For genomic DNA, 20–50 ng typically works, though testing dilutions can help optimize amplification and reduce non-specific products.*
4. Perform PCR using the following thermal cycling conditions: Initial denaturation at 98 °C for 30 s, followed by 35 cycles of denaturation at 98 °C for 10 s, annealing at X ^o^C for 30 s (where X is the primer pair’s calculated annealing temperature), and extension at 72 °C for 30 s per kb, with a final extension at 72 °C for 5 min. Keep the PCR products on ice before use or store at −20 °C for later use.
5. Run agarose gel electrophoresis to verify the amplicon size and purify it as described in Section 3.2.2, Steps 2 and 3.

##### 3.2.3.2 Vector DNA preparation

1. Digest the pEA186 or pKL2310 vector (Supplementary Figure S1) using restriction enzymes *Pst*I and *Xho*I by mixing the following in a microcentrifuge tube: 38 μL of sterile nuclease-free water, 5 μL of 10x CutSmart® Buffer, X μL of vector DNA (up to 5 μg), 1 μL of *Pst*I (10 units/μL) and 1 μL of *Xho*I (10 units/μL). Gently mix by pipetting, briefly centrifuge, and incubate at 37 °C for 12 to 18 h.
*Expected fragment sizes for pEA186 are 16,942 bp, 454 bp, and 431 bp, and for pKL2310 are 15,068 bp and 56 bp.*
2. Run agarose gel electrophoresis and purify the linearized vector DNA (~17 kb for pEA186 and ~15 kb for pKL2310) as described in Section 3.2.2, Steps 2 and 3.

##### 3.2.3.3 Assemble vector and insert DNAs

1. Assemble the linearized vector DNA and insert DNA fragment using Gibson assembly.
2. Example 1: Cloning of a 3 kb insert fragment (20 ng/μL) into the 15 kb *Xho*I and *Pst*I digested, gel-purified pKL2310 (60 ng/μL) using Gibson Assembly as follows. In a 1.5 mL microcentrifuge tube, set up a 10 μL reaction by mixing 1.33 μL of sterile nuclease-free water, 1.67 μL of vector DNA (0.01 pmol), 2.0 μL of insert DNA (0.02 pmol), and 5 μL of NEB HiFi DNA Assembly Master mix. Mix well, briefly centrifuge, then incubate the reaction mix at 50 °C for 15–60 min in a thermocycler or water bath.
3. Example 2: Cloning of a 3 kb insert fragment (20 ng/μL) into the 15 kb *Xho*I and *Pst*I digested, gel-purified pKL2310 (60 ng/μL) using directional ligation as follows. In a 1.5 mL microcentrifuge tube, set up a 10 μL reaction by mixing 3.33 μL of sterile nuclease-free water, 1 μL of T4 DNA ligase buffer, 1.67 μL of vector DNA (0.01 pmol), 3.0 μL of insert DNA (0.03 pmol), and 1 μL of T4 DNA ligase. Mix well, briefly centrifuge, then incubate the reaction mix at room temperature (22 °C) for 30–60 min.
4. After completing the Gibson assembly or ligation reactions, proceed with *E. coli* transformation, plasmid isolation, and construct confirmation following the protocol outlined in Section 3.2.2, Steps 7 to 11.

### 3.3 Introduction of INTEGRATE vectors into *Agrobacterium*

The INTEGRATE vectors can be introduced into *Agrobacterium* cells using either electroporation or the freeze–thaw method (Wise et al., 2006). Electroporation is preferred for its high efficiency (up to 10^6^ vs. 10^2^–10^3^ transformants/μg DNA for freeze–thaw), making it ideal for introducing INTEGRATE vectors. This method applies high intensity electric field pulses to create transient membrane pores for DNA uptake (Dower et al., 1988). Electroporation has been successfully used to deliver plasmids up to 200 kb (Wise et al., 2006). In contrast, the freeze–thaw method (Chen et al., 1994) relies on membrane disruption during temperature cycling. Although less efficient, the freeze–thaw method is simple, inexpensive, and particularly suitable for laboratories without access to an electroporator and expensive consumables (e.g., disposable electroporation cuvettes) and for routine transformations when high efficiency is not critical. Here we describe both methods; the choice should be guided by transformation efficiency requirements, plasmid size, and available resources.

#### 3.3.1 Electro-competent cell preparation and transformation

1. To prepare electrocompetent *Agrobacterium* cells from a 50 mL culture, grow a seed culture by inoculating a single colony of the desired *Agrobacterium* strain into 5 mL of LB broth in a 50 mL conical tube. Incubate at 28 °C with shaking at 200 rpm for 12–16 h until the culture reaches stationary phase. The next day, add 5 mL of the seed culture into 50 mL of fresh LB broth (1:10 dilution) in a 250 mL flask. Incubate at 28 °C with shaking at 200 rpm for 3–4 h or until the culture reaches early stationary phase with a cell density (OD_600_) of 1.2 ~ 1.8.
2. Incubate the culture on ice for 20–30 min, then transfer it to a pre-chilled 50 mL sterile centrifuge tube. Centrifuge at 4,000 × g for 10 min at 4 °C and discard the supernatant. Wash the pellet with 50 mL of ice-cold sterile water, centrifuge again, and discard the supernatant. Repeat this washing step three more times, gently resuspending the pellet each time by pipetting—do not vortex, as this may reduce cell viability and transformation efficiency. After the final wash, resuspend the pellet in 5 mL of sterile, ice-cold 10% glycerol, centrifuge again, and discard the supernatant. Finally, resuspend the pellet in 500 μL of ice-cold 10% glycerol and aliquot 40 μL into pre-chilled 1.5 mL microcentrifuge tubes. Flash-freeze the aliquots in liquid nitrogen and store at −80 °C until use.
*A key to preparing electrocompetent cells is thorough salt removal to create a low-ionic environment, which is essential to prevent arcing and enhance survival during electroporation.*
3. For *Agrobacterium* transformation (Supplementary Figure S3), add 1 μL of confirmed INTEGRATE plasmid DNA (0.1–0.3 μg) to 40 μL electrocompetent cells. Mix gently and transfer to a prechilled, 0.2 cm-gap electroporation cuvette. Keep the cuvette on ice while setting the electroporator to 2.5 kV, 25 μF capacitance, and 400 *Ω* resistance, or use the preset *Agrobacterium* program if available (e.g., Bio-Rad Gene Pulser).
*During Agrobacterium transformation, optimal electroporation conditions are critical for success. When adding the cell-DNA mixture to the cuvette, ensure the suspension settles evenly at the bottom and contacts both metal plates uniformly. If needed, gently tap the cuvette on a flat surface to distribute the mixture. Before pulsing, confirm that the cuvette’s metal contacts are clean and make full contact with the electroporator electrodes—this ensures proper conductivity and maximizes DNA uptake efficiency.*
4. Wipe the outside of the cuvette with a Kimwipe to remove condensation moisture, then insert it into the electroporator. Deliver a single electric pulse and immediately return the cuvette to the ice to stabilize the cells. Add 500 μL SOC or LB medium to the cuvette and gently pipette to resuspend cells. Transfer the cells to a 2 mL microcentrifuge tube, seal the lid with parafilm, and lay the tube horizontally for optimal aeration. Incubate at 28 °C with shaking for 2 h.
  *To enhance post-electroporation cell recovery and improve transformation efficiency, it is important to add a suitable growth medium to the cells IMMEDIATELY after electroporation. This step promotes rapid recovery, ensuring that the transformed cells survive and express genes from the introduced DNA. Using larger size culture tubes* (e.g.*, 14 mL round bottom tubes*) *and 1 mL outgrowth medium can enhance transformation efficiency.*
5. Plate 100 μL of the culture onto a YEP plate containing appropriate antibiotics and incubate at 28 °C for 36–48 h to allow colony formation.

#### 3.3.2 Freeze-Thaw competent cell preparation and transformation

1. To prepare Freeze–Thaw (FT) competent *Agrobacterium* cells from a 50 mL culture, begin with a seed culture as described in Section 3.3.1, Step 1.
2. Incubate the culture on ice for 20–30 min, then transfer to a pre-chilled 50 mL sterile centrifuge tube. Centrifuge at 4,000 × g for 10 min at 4 °C. Discard the supernatant and gently resuspend the pellet in 1 mL ice-cold sterile 20 mM CaCl_2_. Aliquot 100 μL into pre-chilled 1.5 mL tubes. Flash-freeze in liquid nitrogen and store at −80 °C.
3. For transformation, add 1–5 μL of confirmed INTEGRATE plasmid DNA (0.1–1 μg) to 100 μL thawed FT competent cells. Mix gently and keep on ice.
4. Freeze the mixture in liquid nitrogen for 10 s using long-handled tweezers, then put the tube in a 37 °C water bath for 5 min. Add 1 mL SOC or LB medium and transfer to a 2 or 14 mL tube, mix well, and incubate at 28 °C with shaking (200 rpm) for 2 h.
5. Centrifuge at 10,000–15,000 rpm for 2 min. Discard ~1 mL of the supernatant and resuspend the pellet in the remaining volume. Plate the culture onto a YEP plate with appropriate antibiotics and incubate at 28 °C for 36–48 h to allow colony formation.

### 3.4 Isolation and analysis of INTEGRATE insertion and deletion mutants

After *Agrobacterium* transformation and recovery, cells are plated on selective solid media. Although transposition can occur in liquid culture, plating reduces competition within mixed populations (Vo et al., 2021; Aliu et al., 2022; Gelsinger et al., 2024). Due to the high transformation efficiency, selection relies primarily on antibiotic resistance conferred by the vector. For pEA186 (Supplementary Figure S1), which carries an RFP marker, transformed colonies can also be visually identified – typically >99% after electroporation. However, RFP expression alone only confirms plasmid presence, not successful transposon insertion, as RFP can be expressed from the vector without integration. Therefore, colony PCR using genome-specific primers is essential to verify insertional mutagenesis. Our screening consistently shows genetic heterogeneity among primary colonies, which are formed after *Agrobacterium* transformation, often revealing mixed or non-insertional PCR products (Aliu et al., 2022). This suggests that transposition lags behind cell division, allowing the propagation of multiple alleles in one colony (Vo et al., 2021; Aliu et al., 2022; Gelsinger et al., 2024). To obtain clonal targeted insertion, we recommend a colony purification and PCR screening step before the downstream analyses (see Figure 3A-3).

#### 3.4.1 Colony purification and PCR screening

1. Scrape all primary transformant colonies (Figure 3A-3) and resuspend in 5 mL YEP medium with appropriate antibiotics. Incubate at 28 °C with shaking (200 rpm) for 12–16 h. Perform serial dilutions up to 10^6^-fold and plate 50–100 μL of the final dilution onto YEP plates with the appropriate antibiotic. Incubate at 28 °C for 48 h to allow transposon insertions to become fixed within individual colonies. Screen resulting colonies by colony PCR to confirm integration.
2. INTEGRATE-mediated transposon insertion in *Agrobacterium* typically occurs 48–52 bp downstream of the protospacer, with occasional insertions up to 80 bp from the protospacer (Aliu et al., 2022). To ensure comprehensive detection, design a reverse primer at least 200 bp downstream of the target site. Because the first ~50 bases of Sanger sequencing reads are often low quality, design the forward primer or sequencing primer at least 150 bp upstream of the crRNA target site.
*For large DNA fragment deletion assays, forward primer of the first target (target 1) and reverse primer of the second target (target 2) are essential for validating deletion events. To ensure efficient amplification, all screening primers should have melting temperatures (Tm) within 5 °C of each other. We also recommend using the Primer3 tool (such as NCBI Primer-BLAST) for optimal primer design, as it offers detailed insights into potential off-target binding sites.*
3. Pick a colony using a sterile 10 μL pipette tip and resuspend in 10–20 μL of sterile water. Gently resuspend *Agrobacterium* cells by pipetting.
4. On ice, set up 20 μL PCR reactions in 0.2 mL PCR tubes by mixing 5.2 μL of sterile nuclease-free water, 10 μL of 2x Taq DNA polymerase master mix, 0.4 μL of custom forward primer (final concentration 0.2 μM), 0.4 μL of custom reverse primer (final concentration 0.2 μM), and 4 μL of resuspended cell suspension (PCR template).
*To ensure reliable interpretation of colony PCR results, include appropriate controls for PCR screening. Genomic DNA from wild-type strains serves as an ideal negative control. If unavailable, contamination-free wild-type colonies may be used instead.*
5. Perform PCR using the following thermal cycling conditions: Initial denaturation at 94 °C for 1 min, followed by 35 cycles of denaturation at 94 °C for 30 s, annealing at X ^o^C for 30 s (where X is the primer pair’s calculated melting temperature Tm-based annealing temperature), and extension at 68 °C for 1 min per kb, with a final extension at 68 °C for 5 min. Keep the PCR products on ice until further use or store at −20 °C for later use.
6. Confirm the PCR product via gel electrophoresis as described in Section 3.2.2, Steps 2.
7. After confirming homogeneous colonies with targeted insertion, purify PCR products using spin column-based kits (e.g., QIAquick PCR purification kit) or PCR product clean-up reagents (e.g., ExoSAP-IT™ PCR clean-up reagent), following the manufacturer’s instructions.
8. Sequence the purified PCR products using a preferred Sanger sequencing service provider to confirm the targeted insertion. Precisely map the cargo DNA insertion site using recommended software tools (see Materials section).

#### 3.4.2 Screening of targeted DNA integration

1. As outlined in Section 3.1.2, vector pEA186 – carrying an RFP cassette as the mini-Tn cargo – can be adapted for targeted DNA integration, such as gene insertion for expression, or insertional mutagenesis. Confirmation of targeted DNA integration requires colony PCR using genome-specific primers (see Section 3.4.1, Steps 4 to 7). INTEGRATE generates two main insertion types (Figure 3B-7): T-RL (right end adjacent to target) and T-LR (left end adjacent due to cargo inversion). Orientation is determined using PCR with genome- and cargo-specific primers (Figure 2A) or confirmed by Sanger sequencing. If the mini-Tn cargo size is large (>3 kb), cargo-specific primers are paired with the genome-specific primers to verify the targeted insertion as well as the Tn cargo orientation. For example, if a reverse cargo-specific primer is paired with a forward genomic primer and produces an amplicon, this indicates a T-RL orientation. Conversely, amplification with a reverse genomic primer suggests a T-LR configuration.
2. A successful targeted DNA integration is indicated by an increased PCR amplicon size compared to the wild-type control. For example, the mini-Tn cargo in pEA186 is 1.6 kb; if the wild-type control yields a 0.3 kb product, a successful insertion should produce a 1.9 kb fragment (0.3 kb + 1.6 kb) (Figure 3B).
  *INTEGRATE-mediated transposition is characteristically accompanied by the formation of a 5 bp target site duplication* (TSD; Peters et al., 2017)*. This occurs when the transposition machinery introduces staggered double-strand breaks at the genomic target site, resulting in 5-nucleotide overhangs on either side of the insertion site. During the integration of the transposon, the host’s DNA repair system fills in these overhangs, duplicating the flanking 5 bp sequence on both sides of the transposon. The presence of this hallmark duplication serves as a molecular signature of successful transposition and can aid in the confirmation of insertion events.*
3. Once the candidate colony is confirmed by Sanger sequencing (see Section 3.4.1, Step 7), streak it onto a YEP plate with appropriate antibiotics. Then proceed with INTEGRATE vector eviction as described in Section 3.5.

#### 3.4.3 Generating and screening of targeted DNA deletion

INTEGRATE-mediated genomic deletion in *Agrobacterium* requires a second step involving site-specific recombination (Figure 3C). First, INTEGRATE vector (e.g., pKL2310, Supplementary Figure S1, or its derivative) is used to insert *loxP* sequence at two target sites of the *Agrobacterium* genome. A second plasmid carrying the *Cre* recombinase (e.g., pKL2315; Supplementary Figure S1) is then introduced into the *loxP*-tagged strain. Upon expression, Cre mediates recombination between the two *loxP* sites, excising the intervening genomic DNA (Kuhn and Torres, 2002). The orientation of *loxP* sites is critical – direct repeats enable deletion, while inverted repeats may cause inversion or duplication (Supplementary Figure S4). Therefore, the orientation of two *loxP* sites (Figure 3B-7) must be confirmed by Sanger sequencing before introducing the Cre recombinase vector.

1. As described in Section 3.1.2, vector pKL2310 is specifically designed for targeted DNA deletion, carrying a *loxP* site within its mini-Tn cargo. Two spacer sequences targeting the desired genomic sites can be cloned into the crRNA array (see Section 3.2.2). After introducing the INTEGRATE construct into *Agrobacterium* (see Section 3.3. *The efficiency of the electrocompetent cells used can significantly influence the outcome of targeted deletions*), colonies are screened and validated by PCR using target-specific genomic primers (see Section 3.4.1), followed by Sanger sequencing to confirm *loxP* insertions and determine their orientation (see Section 3.4.1).
  *The INTEGRATE system displays transposon immunity, where an initial insertion blocks further insertions at or near the same site. This can hinder small-fragment deletions. However, we achieved a 2.5 kb deletion in* Agrobacterium *by simultaneously inserting both* loxP *sites using a multiplexed CRISPR array, followed by two rounds of colony purification. Stepwise insertions should be avoided, as immunity makes this approach ineffective.*
2. The *Agrobacterium* colony confirmed to carry *loxP* sites at both target sites with the same orientation is used to prepare electrocompetent cells (see Section 3.3) for transformation with the Cre recombinase plasmid pKL2315.
  *Although off-target INTEGRATE insertions are rare, we strongly recommend whole-genome sequencing before Cre expression if resources are available. Multiple identical recombination sites can cause unpredictable outcomes. Low-frequency off-target events reported in E. coli* (Vo et al., 2021) *highlight the need for thorough genomic validation.*
3. Transform *loxP*-tagged *Agrobacterium* electrocompetent cells with the Cre-expressing plasmid pKL2315 using the steps described in Section 3.3 (Figure 3C; Supplementary Figure S3). Incubate transformed cells on YEP plates containing 50 mg/L kanamycin to select for pKL2315.
4. Perform colony purification on resulting colonies (see Section 3.4.1, Step 1).
5. Conduct colony PCR as described in Section 3.4.1 Steps 3 to 5 using target-specific primers (Figure 3C-9): (1) target 1 forward and target 2 reverse primers to detect deletion events, and (2) target 1 forward/reverse or target 2 forward/reverse primers to detect the presence of target DNA fragment.
6. Verify the deletion mutations using 1% agarose electrophoresis as described in Section 3.4.1, Step 6. A pure deletion mutant colony must have a fragment amplified by the target 1 forward and target 2 reverse primers only, whereas a mixed population colony will also have fragments amplified by target 1 forward/reverse or target 2 forward/reverse primer sets (Figure 3C-9). Confirm the deletion by Sanger sequencing.
  *Due to the heterogeneous nature of INTEGRATE mutagenesis, it is essential to confirm that the deletion mutant is clonal, not a mixed population. Use PCR screening to verify the absence of original dual* loxP *sites in the target sites.*
7. If deletion is not achieved or all the screened colonies are mixed populations, then perform colony purification step once more and repeat the PCR screening steps (see Section 3.4.3, Steps 5 and 6).
8. If mixed insertion bands of equal intensity continually persist, purify the colony further by streaking cultures onto YEP agar plates containing 50 mg/L kanamycin. Pure deletion mutants can typically be obtained through two to three sub-streaking steps.

### 3.5 Post-engineering eviction of INTEGRATE vectors

After genome modification, removal of the INTEGRATE vector is essential to minimize metabolic burden and maintain genetic stability in *Agrobacterium*. Retention of unnecessary plasmid DNA can compromise strain performance and long-term stability (Silva et al., 2012). To ensure a clean, engineered background, we use two primary curing strategies: SacB-mediated counterselection and plasmid incompatibility mediated eviction, described below.

SacB-mediated curing is an highly efficient strategy. The *sacB* gene encodes levansucrase, which converts sucrose into toxic levan (Gay et al., 1983; Steinmetz et al., 1983). INTEGRATE vectors such as pEA186 and pKL2315 carry *sacB* and can be selectively eliminated by culturing cells without antibiotics, followed by plating on 5% sucrose medium. Cells retaining the plasmid die due to levan accumulation, while plasmid-free segregants survive.

Plasmid incompatibility exploits competition between plasmids with similar replication and partitioning systems. Introducing a second plasmid with an incompatible origin forces a replication conflict, resulting in a gradual loss of the resident INTEGRATE vector. This approach is particularly effective when selective conditions are applied to maintain only the preferred plasmid. In the INTEGRATE-mediated deletion experiments, pKL2310 (*loxP* delivery vector) is evicted by introducing pKL2315 (Cre expression vector). Both vectors share the same pVS1 ORI but differ in antibiotic resistance. Selecting kanamycin resistance promotes the retention of pKL2315. Loss of pKL2310 can be confirmed by spectinomycin sensitivity.

#### 3.5.1 Plasmid eviction through SacB-mediated sucrose counterselection

1. Once the insertion or deletion of targeted genes is confirmed, the INTEGRATE vector (pEA186 or its derivatives) and the Cre-expressing plasmid (pKL2315) can be removed from the engineered *Agrobacterium* strains using the SacB-mediated counterselection strategy.
2. Inoculate the *Agrobacterium* strain carrying the plasmid to be evicted into liquid YEP medium without antibiotics. Incubate at 28 °C with shaking at 200 rpm for 12–16 h.
3. Perform a 1:10 serial dilution of the overnight culture. Plate 100 μL of dilution onto YEP agar supplemented with 5% sucrose (no antibiotics). Incubate at 28 °C for 48 h.
  *For SacB-mediated plasmid curing, directly streaking overnight cultures onto sucrose-containing agar plates offers a convenient alternative to serial dilution and plating. Alternatively, a confirmed* Agrobacterium *colony with the correct genomic edit can be streaked directly onto sucrose plates, bypassing liquid culture step. However, this method is generally less effective, as it often yields fewer surviving colonies.*
4. Identify successfully cured mutants by comparing growth on selective and non-selective media. Pick multiple colonies and spot them onto YEP plates with and without plasmid-specific antibiotics. True cured colonies will grow only on non-selective plates, while those retaining the plasmids will survive on selective media. Additional validation can be done by inoculating putative cured mutants into liquid media with and without antibiotics. Lack of growth in selective media confirms successful plasmid curing.
  *False positives during SacB-mediated curing can result from: (1) mutations in the sacB gene; (2) plasmid rearrangements deleting* sacB *while retaining other elements; (3) leaky* sacB *expression or inherent sucrose tolerance in some* Agrobacterium *strains; and (4) insufficient sucrose concentrations that fail to exert effective selective pressure. To minimize false positives, we recommend using 5–10% sucrose for strong counterselection and pairing* sacB *with a strong promoter to ensure robust expression.*
5. Prepare glycerol stocks of engineered *Agrobacterium* strains. Inoculate a colony into 5 mL of LB or YEP medium containing appropriate antibiotics in a 50 mL conical tube. Incubate at 28 °C with shaking (200 rpm) for 16–18 h. Mix 500–650 μL of the overnight culture with an equal volume of sterile 60% glycerol in a labeled screw-cap cryovial. Thoroughly mix the tubes and store at −80 °C for long-term preservation.

#### 3.5.2 INTEGRATE vector curing via plasmid incompatibility

1. In *Agrobacterium* genomic deletion experiments, the INTEGRATE vector pKL2310 is removed using plasmid incompatibility after confirming the correct insertion orientations of the two *loxP* sites. If a pKL2310-based vector is used for targeted DNA insertions, it can be removed by another *sacB*-carrying vector with pVS1 ORI (such as pKL2315).
2. Following the steps in Section 3.4.3, first prepare competent cells (see Section 3.3), introduce the Cre-expression vector pKL2315, and incubate the transformed bacteria on YEP plates with 50 mg/L kanamycin to select for pKL2315. Kanamycin selection ensures preferential retention of pKL2315 over pKL2310, which confers spectinomycin resistance. Successful eviction of pKL2310 can be confirmed by culturing the transformed colonies in a liquid YEP medium supplemented with 100 mg/L spectinomycin, where susceptibility indicates the loss of the original vector, pKL2310. The plasmid used for the curing, pKL2315, then can be removed via SacB-mediated sucrose counterselection as described in Section 3.5.

## 4 Results

In this work, we demonstrate the use of the INTEGRATE vector pKL2310 and Cre-recombinase vector pKL2315 to generate a disarmed *Agrobacterium rhizogenes* K599 strain via targeted deletion of the T-DNA region. Using a pEA186-based INTEGRATE vector, we also demonstrate how to generate a thymidine auxotrophic mutant using the disarmed K599 strain.

### 4.1 Disarming *A. rhizogenes* K599

We used a two-step procedure to disarm *Agrobacterium rhizogenes* K599 strain, which carries a 202 kb root inducing plasmid pRi2659 (Figure 4A). *A. rhizogenes* strain K599 has been widely used for hairy root induction and its 5.48 Mb genome carries three replicons: a circular chromosome, a linear chromid, and pRi2659 (Caspi et al., 2020). To delete the ~ 15 kb T-DNA region, we first inserted *loxP* into two target sites just outside of the left (LB) and right border (RB) sequences, so that Cre-mediated recombination can delete the entire T-DNA region, including both border sequences (Figure 4B). Designed oligonucleotide sequences for the crRNAs are shown in Figure 4C and Example 2 of Section 3.2.1.

After cloning the crRNAs into *Bsa*I-digested pKL2310 (Figure 4D; Section 3.2.3), the INTEGRATE vector was further modified for gentamicin selection due to the resistance of K599 to spectinomycin. The spectinomycin resistance gene cassette in pKL2310 was replaced with the gentamicin resistance gene from pEA244 (Aliu, 2025) using two restriction enzymes *Spe*I and *Xma*I. The final construct (pLC115) was introduced into wild-type *A. rhizogenes* K599 strain via electroporation. After the colony purification step, PCR screening was performed using the target-specific primer sets (LB-F & LB-R; RB-F & RB-R) to identify homogeneous colonies with targeted insertion (Supplementary Figure S5; Sections 3.3 and 3.4). During the first screening, two out of twelve (2/12) colonies showed targeted insertion at the RB target site, while no targeted insertion was detected at the LB target site. After one more round of colony purification step using the two colonies with RB target insertion, one out of twelve (1/12) colonies showed partial insertion (i.e., mixed population) at the LB target site. After another round of colony purification step, eleven out of twelve (11/12) colonies had targeted insertion at both RB and LB target sites (Supplementary Figure S5).

Sanger sequencing analysis revealed that *loxP* site was inserted at 49 bp downstream from the protospacer of the LB target with a precise 5-bp TSD (Figure 5A, left panel). Likewise, another *loxP* site was inserted 48 bp downstream of the RB target protospacer (Figure 5A, right panel). In both target sites, *loxP* Tn-cargo had the T-RL orientation; thus, the mutant was used for Cre-mediated deletion (Supplementary Figure S4). As illustrated in Figure 5B, introduction of the Cre-recombinase vector pKL2315 resulted in precise T-DNA deletion, leaving one *loxP* Tn-cargo and a 5-bp TSD from the RB target site. After the eviction of pKL2315 by *sacB*-mediated counterselection (Section 3.5.2), the disarmed K599 strain (K599ΔT-DNA = K599dT) was further validated by whole genome sequencing (Supplementary Figure S6).

As a final verification step, we performed hairy root induction assay using *Kalanchoe* plants. WT and K599dT strains were transformed with pLC112K (Supplementary Figure S7), which carries betalain biosynthesis marker *RUBY* (He et al., 2020), so that the transgenic hair roots can be identified by vivid purple color. WT and K599dT strains were inoculated into the stem of 4-5-week-old Kalanchoe plants and hairy root production was monitored for the next 3 weeks. As shown in Figure 5C, only the WT strain produced purple hairy roots, whereas neither the disarmed strain K599dT nor the water control generated any hairy roots at the inoculation sites, indicating that we successfully disarmed the K599 strain.

### 4.2 Generating thymidine auxotrophic K599

To generate a thymidine auxotrophic strain, the disarmed K599 was transformed with a second INTEGRATE vector targeting the *thyA* coding sequence (Figure 6A; Example 1 of Section 3.1.2). Oligonucleotides for the crRNA spacer (Supplementary Table S1; Example 1 of Section 3.1.2) were cloned into INTEGRATE vector pEA244 (Aliu, 2025), which has a gentamicin resistance gene for bacterial selection and *tonB* terminator (Postle and Good, 1983) as a cargo, generating pKL2656. The strain K599dT was transformed with pKL2656 as described Section 3.3, and after bottlenecking and colony purification steps (Section 3.4.1), colonies with targeted insertion were identified and validated by Sanger sequencing (Figure 6B; Section 3.4.2). The mini Tn-cargo was inserted 50 bp downstream from the *thyA* protospacer with T-RL orientation. The resulting strain K599dTT exhibited thymidine-dependent growth (Figure 6C) and sensitivity to gentamicin, confirming successful eviction of pKL2656.

## 5 Discussion

*Agrobacterium*-mediated transformation has long served as a critical tool for plant bioengineering, enabling the stable integration of foreign DNA into plant genomes (Gheysen et al., 2022; Azizi-Dargahlou and Pouresmaeil, 2024). This method has facilitated countless advances in plant genetic engineering, from basic research to crop improvement. Despite its widespread use, only a limited number of *Agrobacterium* strains have been routinely adopted for plant transformation (De Saeger et al., 2021; Goralogia et al., 2025). The narrow diversity of strains used in transformation protocols restricts the potential for optimization and innovation in plant bioengineering.

Traditionally, genetic modification in bacteria, including *Agrobacterium*, has relied on homologous recombination (HR) to achieve gene knockouts or insertions. While HR offers high specificity, its efficiency is often low, particularly when targeting chromosomal loci (Lazo et al., 1991; Aliu et al., 2022, 2024). The process becomes even more challenging in strains deficient in the *recA* gene, which is essential for homologous recombination (Lazo et al., 1991; Aliu et al., 2022). These limitations have hindered the development of novel *Agrobacterium* strains with improved transformation capabilities or altered biological properties.

Recent advances in genome engineering technologies have opened new avenues for precise and efficient genetic modification in *Agrobacterium* (Aliu et al., 2022; Bian et al., 2022; Pennetti et al., 2025; Rodrigues et al., 2021). Among these, the CRISPR RNA-guided INTEGRATE system stands out as a transformative tool. INTEGRATE (Insertion of Transposable Elements by RNA-guided Targeting) leverages Type I-F CRISPR-Cas machinery to direct site-specific integration of genetic elements into bacterial genomes without relying on HR (Klompe et al., 2019; Vo et al., 2021; Aliu et al., 2022). This system offers a robust and programmable platform for *Agrobacterium* genome engineering, enabling researchers to overcome the limitations of traditional methods.

Here, we provide a comprehensive guide for INTEGRATE-mediated genome engineering in *Agrobacterium*. We detail protocols, optimization strategies, and troubleshooting tips to facilitate the adoption of this technology by researchers aiming to develop novel strains for plant transformation or conduct functional genomics studies. From crRNA design and oligonucleotide synthesis to vector construction and eviction, each step is carefully outlined to ensure reproducibility and efficiency (see Table 1 for a detailed troubleshooting guide).

**Table 1:** Troubleshooting guide.

Issues	Possible cause	Recommended solution
Step B: INTEGRATE Vector Modification		
Low cloning efficiency	crRNA spacer oligos lack 5′ phosphate groups	Phosphorylate oligos prior to ligation.
	Poor INTEGRATE vector DNA quality after agarose gel electrophoresis	INTEGRATE vectors are large (>15 kb) and users may experience low recovery from the agarose gel resulting in low concentration. Use a commercial gel extraction kit according to the manufacturer’s instruction to prepare high-quality pure DNA for ligation or Gibson assembly reactions. Verify 260/280 (≥1.8) and 260/230 (≥2.0) ratios before use.
	Inadequate vector-insert contact	During ligation, ensure thorough mixing of vector backbone and inserts/oligos first before adding other reagents into ligation mix.
	Recombination between Vch repeats	Ensure oligos are properly designed as described in Step B1.
	Inefficient ligation with multiple oligos	Use more T4 DNA ligase enzyme or extend the ligation time.
	Poor *E. coli* competent cells	Use high-efficiency competent cells from commercial vendor.
	Poor enzymes	T4 DNA ligase or Gibson assembly enzyme mix can be compromised. Use verified enzyme source.
Steps C and D: *Agrobacterium* Transposition Assay		
No or few colonies formed after transformation	Toxicity from overexpression of transposon components	Replace strong constitutive promoters with weaker or inducible alternatives.
	Plasmid backbone incompatibility for target strain	Use a validated vector backbone compatible with the target strain.
	Low *Agrobacterium* competent cell efficiency	Use high-efficiency *Agrobacterium* competent cells (see Steps C1 and C2). Electroporation recommended over the freeze–thaw method, due to its superior efficiency.
	Low quality plasmid DNA	Use clean intact plasmid DNA. Verify plasmid DNA by agarose gel electrophoresis after restriction enzyme digestion.
Poor colony isolation after colony purification	Overcrowded plating	Perform serial dilutions (e.g., up to 10^−6^) and plate 50–100 μL for better isolation.
Non-specific PCR or absence of DNA band	Suboptimal PCR conditions	Optimize PCR conditions prior to screening. Include a positive-control such as genomic DNA from wild-type strain. Targeted insertion can significantly increase the amplicon size, thus extension time should be extended to complete the amplification step.
	PCR contamination	Include a no-template control to check contamination.
Low transposition efficiency: no or partial insertions	Low efficiency crRNA(s)	Prolong the culture time or repeat subculture in liquid medium before serial dilution and spreading on solid selective medium (Step D1). If no targeted insertion detected after extra rounds of colony purification steps, design new crRNAs. If targeted insertion is not detected from all target sites (i.e., partial insertion), then grow the colonies with partial insertion and repeat colony purification steps (see Steps D1 and D2). If necessary, redesign crRNAs for target sites that have no insertions.
	Target immunity due to existing Tn-cargo or Tn7-like transposons	Targeted insertion of a Tn-cargo into the proximity (< 5 kb) of existing cargo or other Tn7-like transposons can be challenging due to target immunity. Select different target sites. For small sequence deletion (<5 kb), insert two loxP sites simultaneously to avoid target immunity.
	Low plasmid copy number	Plasmid copy number has a dosage effect on both INTEGRATE component expression and Tn-Cargo copy numbers. Using a high copy number plasmid backbone can increase transposion efficiency.
	Weak promoter	Similar to plasmid copy number, strong constitutive or inducible promoters can enhance overall expression of the INTEGRATE system.
Unsuccessful DNA deletion	Unintended recombination events	Perform whole-genome sequencing before Cre recombinase-mediated recombination to check for off-target insertion sites.
	Heterogeneous INTEGRATE mutagenesis	Use colony PCR to confirm loss of original loxP sites; verify presence of deletion band using target 1 forward and target 2 reverse primers.
	Low efficiency for large targeted deletions	After colony purification step, inoculate a few single colonies and subculture every 24 h. After 48 h, use culture for colony PCR. Continue subculture if desired targeted deletion is not obtained.
Ambiguous PCR results	Lack of proper controls	Include proper controls (positive and negative). E.g wild-type genomic DNA or colony.
Poor-quality or ambiguous Sanger sequencing chromatograms	mixed population with varying integration patterns or orientations	Re-streak colonies on solid media to isolate single colonies; repeat PCR and sequencing to confirm clonal purity.
Step E: Post-Engineering Evection of INTEGRATE Vectors		
False positives	Residual plasmid retention	Spot colonies on selective and non-selective plates; cured clones grow only on non-selective media. For Cre/loxP-mediated DNA deletion, the loxP INTEGRATE vector is evicted via plasmid incompatibility. A short culture time after introducing the Cre recombinase vector (pKL2315) may result in persistance of the loxP INTEGRATE vector in some colonies. Check antibiotics sensitivity for both Spectinomycin (loxP INTEGRATE) and Kanamycin (Cre recombinase). Use colonies that only grow on 5% sucrose medium without Spectinomycin or Kanamycin.
	sacB mutations or low sucrose concentration	Check the sucrose sensitivity using the primary transformants, which should only grow on selective medium without 5% sucrose. If necessary, use 10% sucrose for stronger selection or use a stronger promoter for sacB expression.

One of the most compelling features of the INTEGRATE system is its capacity for multiplexed gene editing. Researchers can simultaneously target multiple genes, including redundant or functionally related loci, to dissect complex regulatory networks. By inactivating several genes in parallel, researchers can uncover subtle phenotypic effects and gain insights into bacterial physiology and virulence mechanisms (Aliu et al., 2024; Lopez-Agudelo et al., 2025). However, it needs to be noted that the maximum number of crRNAs and Tn-cargo size for efficient editing should be determined before extensive applications. For instance, while we have observed highly efficient targeted insertions into up to four different intergenic regions, targeting essential coding regions tends to yield variable outcomes. Additionally, the position of crRNAs within the CRISPR array can influence the overall targeting efficiency as the distal crRNAs often result in lower efficiencies (Aliu et al., 2022). In general, the targeting efficiency tends to decrease as the Tn-cargo size increases, especially for those over 5 kb. While the maximum cargo size has yet to be determined, we have successfully inserted multiple Tn-cargos over 10 kb by optimizing crRNA design and performing extra colony purification steps.

Beyond targeted knockouts, the INTEGRATE system also holds promise for genome-scale screening applications. Although not covered in this protocol, the system can be adapted to deliver libraries of crRNAs, enabling high-throughput functional genomics studies. Similar approaches have been successfully implemented in other CRISPR-based systems to interrogate gene function across entire genomes (Sanjana et al., 2014; Bock et al., 2022). Such strategies could be instrumental in identifying novel genes involved in plant-microbe interactions, stress responses, or metabolic pathways.

The versatility of the INTEGRATE system is further enhanced when combined with site-specific recombinases such as Cre/*loxP*. This combination enables the engineering of chromosome structural variants, including inversions, deletions, and translocations. These modifications can be used to generate isogenic strains that differ only in chromosome architecture, providing a powerful framework to study bacterial chromosome evolution (Aliu, 2025). Using this approach, our group (Aliu, 2025) manipulated the chromosome structure of *Agrobacterium tumefaciens* C58 wild type strain (WT), which harbors two chromosomes – a circular chromosome and a linear chromid, as well as a single chromosome variant (C58F) formed by the natural fusion of the circular and linear chromosomes (Liao et al., 2022). We circularized the linear chromid in the WT strain and the fused linear chromosome in the C58F strain, generating novel variants with distinct genomic architecture. Interestingly, strains with a single chromosome were more competitive and stress-tolerant compared to the WT strain but showed reduced virulence toward plants. These findings suggest that the native chromosome structure of C58 may have evolved to optimize its role as a phytopathogen, balancing fitness and pathogenicity (Aliu, 2025).

These studies highlight the utility and potential of the INTEGRATE system in *Agrobacterium* research. By enabling precise and stable genetic modifications, INTEGRATE facilitates the development of customized strains tailored for specific applications in plant biotechnology. Whether the goal is to enhance transformation efficiency, reduce virulence, or explore fundamental aspects of bacterial biology, this system provides a versatile and accessible toolkit. It is worth noting that there might be strain-specific challenges when implementing the INTEGRATE system, such as preexisting antibiotics resistance requiring vector modifications prior to use. For instance, *Agrobacterium* strain EHA101 is resistant to kanamycin, thus the antibiotics resistance marker gene in the Cre recombinase vector pKL2315 must be replaced with another compatible selection marker, e.g., gentamicin resistance gene. Furthermore, some *Agrobacterium* strains carry extra plasmids, which might be incompatible with the ORIs of the INTEGRATE vectors. In such cases, testing other validated plasmid backbones (ORIs) is highly recommended.

While this protocol is specifically optimized for *Agrobacterium* species, the underlying principles and methodologies of the INTEGRATE system are broadly applicable to other bacterial genera. With appropriate optimization, such as tailoring crRNA design, vector compatibility, and transformation conditions, this system can be adapted for genome engineering in a wide range of bacteria. This flexibility opens new possibilities for functional genomics, synthetic biology, and strain development in both model and non-model bacterial systems. As the demand for precise and efficient genetic tools continues to grow across microbiology and biotechnology, the INTEGRATE system is poised to become a valuable platform for bacterial genome manipulation beyond *Agrobacterium*.

The INTEGRATE system offers several compelling advantages that make it a powerful tool for bacterial genome engineering. One of its key strengths is the ability to mediate the insertion of large DNA fragments—up to 10 kilobases—into specific genomic loci with high efficiency. This capacity is particularly valuable for introducing complex genetic circuits, multi-gene operons, or large regulatory elements. Additionally, the system supports multiplexed gene targeting, enabling simultaneous inactivation or modification of multiple genes. This feature is especially useful for dissecting redundant gene functions or engineering complex phenotypes. Another notable advantage is the system’s high specificity, with little-to-no detectable off-target effects, making it a reliable platform for precise genome modifications. However, the INTEGRATE system also has limitations. It is not well-suited for applications requiring single-nucleotide changes or precise point mutations, which are better addressed by base editors or recombineering approaches. Furthermore, there is a potential risk that previously integrated DNA elements could be remobilized if the cognate transposition machinery is reintroduced or remains active, posing challenges for long-term genomic stability. Despite these limitations, the INTEGRATE system remains a versatile and robust tool for bacterial genome engineering, particularly for applications involving large insertions and multiplexed modifications.

In summary, the CRISPR RNA-guided INTEGRATE system represents a significant advancement in *Agrobacterium* genome engineering. It offers a high-efficiency, multiplexable, and recombination-independent method for genetic manipulation, overcoming many of the limitations associated with traditional HR-based approaches. The protocols and strategies presented in this guide are designed to empower researchers to harness the full potential of INTEGRATE for strain development and functional genomics. As the field of plant biotechnology continues to evolve, tools like INTEGRATE will play a pivotal role in shaping the next generation of *Agrobacterium* strains and unlocking new possibilities in plant genetic engineering.

## Data Availability

The original contributions presented in the study are included in the article/Supplementary material. Further inquiries can be directed to the corresponding authors.

## Supplementary material

SUPPLEMENTARY Figure S1 INTEGRATE vectors for Agrobacterium genome engineering, reprinted from Aliu et al. (2022), licensed under CC BY-NC-ND 4.0.
