# Supplementary material for: IMAGE: INTEGRATE-Mediated Agrobacterium Genome Engineering
Source: Front Microbiol. 2025 Nov 6;16:1676008. doi: 10.3389/fmicb.2025.1676008 (PMC12631759; doi:10.3389/fmicb.2025.1676008)
Supplement: SUPPLEMENTARY Figure S1 — INTEGRATE vectors for Agrobacterium genome engineering, reprinted from Aliu et al. (2022), licensed under CC BY-NC-ND 4.0. [file Data_Sheet_1.pdf]

Supplementary Materials

IMAGE: INTEGRATE-**M**ediated *Agrobacterium* **G**enome **E**ngineering

Ephraim Aliu[1,2,3], Liang-Chun Chen[1,2,3], Keunsub Lee[1,2*], Kan Wang[1,2*]

[1]Department of Agronomy, Iowa State University, Ames, Iowa, USA

[2]Crop Bioengineering Center, Iowa State University, Ames, Iowa, USA

[3]Interdepartmental Plant Biology Major, Iowa State University, Ames, Iowa, USA

[4]Plant Transformation Facility, Donald Danforth Plant Science Center, Saint Louis, Missouri, USA

*Co-Corresponding Author:
Kan Wang, kanwang@iastate.edu; Keunsub Lee, klee@iastate.edu

Solution preparation:

- **100 mg/mL spectinomycin (Sm) solution (1000x stock solution)**
  - Weigh 5.0 g Sm into a clean beaker. Add 50 mL MQ water and dissolve completely
  - Filter sterilize in a 0.22 µm syringe filter.
  - Aliquot (or desired volume) into 1.5 or 2 mL prelabelled Eppendorf tubes.
  - Store at -20°C for 1 year.

- **50 mg/mL kanamycin (Km) solution (1000x stock solution)**
  - Weigh 2.5 g Km into a clean beaker. Add 50 mL MQ water and dissolve completely
  - Filter sterilize in a 0.22 µm syringe filter.
  - Aliquot 1 mL (or desired volume) into 1.5 or 2 mL prelabelled Eppendorf tubes.
  - Store at -20°C for 1 year.

- **50 mg/mL carbenicillin (Cb) (1000x stock solution)**
  - Weigh 2.5 g Cb into a clean beaker. Add 50 mL MQ water and dissolve completely
  - Filter sterilize in a 0.22 µm syringe filter.
  - Aliquot 1 mL (or desired volume) into 1.5 or 2 mL prelabelled Eppendorf tubes.
  - Store at -20°C for 1 year.

- **1% Agarose with RedSafe**
  - Weigh agarose depending on the desired volume (e.g. 1 g in 100 ml 1X TAE for 1%)
  - Dissolve agarose in 1X TAE buffer by heating in a microwave until fully melted.
  - Allow to cool slightly (~6°C),
  - Add RedSafe according to the manufacturer's recommendations (RedSafe is supplied as a 20,000X concentrate. For 100 mL 1X TAE, add 5 µL).
  - Pour into a gel casting tray with an appropriate comb.
  - Allow the gel to solidify before use.

- **Ice cold 10% glycerol (vol/vol)**
  - Measure 10 mL of 100% glycerol into a clean beaker.
  - Add 90 mL of Milli-Q water to achieve a final volume of 100 mL.
  - Mix thoroughly by stirring or vortexing until fully dissolved.
  - Sterilize by autoclaving.
  - Store at 4°C.

- **50% (vol/vol) glycerol**
  - Measure 50 mL of 100% glycerol into a clean beaker.
  - Add 50 mL of Milli-Q water to achieve a final volume of 100 mL.
  - Mix thoroughly by stirring or vortexing until fully dissolved.
  - Sterilize by autoclaving.
  - Store at room temperature for long-term use.

- **60% (vol/vol) glycerol**
  - Measure 60 mL of 100% glycerol into a clean beaker.
  - Add 40 mL of Milli-Q water to achieve a final volume of 100 mL.
  - Mix thoroughly by stirring or vortexing until fully dissolved.
  - Sterilize by autoclaving.
  - Store at room temperature for long-term use.

- **70% (vol/vol) ethanol**
  - Using a granulated cylinder, measure 700 mL of 100% ethanol
  - Add 300 mL of sterile MQ or distilled water
  - Mix well by shaking or inverting the container.
  - Store in a tightly sealed bottle at room temperature.

- **1 L Yeast Extract Peptone (YEP) growth medium**
  - Weigh 10 g yeast extract, 10 g peptone, and 5.0 g NaCl into a clean beaker.
  - Adjust to 1L with MQ water.
  - Autoclave to sterilize.
  - Store at room temperature for long-term use.

- **1 L Luria-Bertani (LB) growth medium**
  - Weigh 25 g of premade LB powder.
  - Adjust to 1L with MQ water.
  - Autoclave to sterilize.
  - Store at room temperature for long-term use.

- **100 mL selective agar plates with antibiotics**
  - Weigh 1.5 g Agar into a clean beaker.
  - Adjust to 100 mL with growth medium (YEP or LB)
  - Autoclave to sterilize.
  - Allow to cool to 50°C - 60°C before adding antibiotics to prevent degradation.
  - Pipette 100 µL of desired antibiotic(s)
  - Gently mix by swirling on a hard surface to prevent bubble formation
  - Pour into sterile Petri dishes (~20-25 mL per plate).
  - Let plates solidify at room temperature.
  - Store at 4°C, protected from light for 4-8 weeks.

- **100 mL 5% Sucrose plates.**
  - Weigh 5 g sucrose and 1.5 g Agar into a clean beaker.
  - Adjust to 100 mL with growth medium (YEP or LB)
  - Autoclave to sterilize.
  - Allow to cool to 50°C - 60°C before adding antibiotics to prevent degradation.
  - Pour into sterile Petri dishes (~20-25 mL per plate).

- Let plates solidify at room temperature,
- Store at 4°C for 4-8 weeks.

- **10X Potassium phosphate Buffer**
  - Weigh 2.31 g of monopotassium phosphate KH_2PO_4 (.i.e 0.17 M) and 12.54 g of dipotassium phosphate K_2HPO_4 (.i.e 0.72 M) into a clean beaker.
  - Add 900 mL MQ water to dissolve.
  - Autoclave to sterilize.
  - After cooling, add 100 mL of sterile 10X phosphate buffer
  - Use for TB preparation.

- **Terrific Broth (TB)**
  - Weigh 12 g of tryptone and 24 g of yeast extract into a clean beaker.
  - Add 100 mL MQ water to dissolve.
  - Add 8 mL of 50% glycerol and mix well
  - Autoclave to sterilize.
  - After cooling, add 100 mL of sterile 10X phosphate buffer
  - Store at 4°C for long-term use.

- **Magnesium (Mg) solution (1 M** $MgCl_2$ / **1 M** $MgSO_4$)
  - In a clean beaker, weigh 20.3 g of $MgCl_2{\cdot}6H_2O$ and 24.7 g of $MgSO_4{\cdot}7H_2O$ for a 1 M $MgCl_2$ and $MgSO_4$ stock solution.
  - Add 100 mL MQ water to dissolve. Place a stir bar in the beaker and stir until completely dissolved.
  - Filter sterilize through a 0.22 µm sterile filter into a sterile prelabelled storage bottle.
  - Store at room temperature for long-term use.

- **1 L Super Optimal Broth (SOB)**
  - Weigh 20 g of tryptone, 5.0 g of yeast extract, 0.5 g NaCl, and 0.125 g KCl into a clean beaker.
  - Add 900 mL MQ water to dissolve. Place a stir bar in the beaker and stir until completely dissolved.
  - Autoclave to sterilize.
  - Cool to room temperature
  - Add 10 mL of sterile Mg solution
  - Bring volume to 1 L with sterile water.
  - Store at room temperature for long-term use.

- **100 mL 50 mM Calcium chloride solution**
  - In a clean beaker, weigh 0.735 g of $CaCl_2{\cdot}2H_2O$
  - Add 100 mL MQ water. Place a stir bar in the beaker and stir until completely dissolved.
  - Pass the solution through a 0.22 µm sterile filter into a sterile prelabelled storage bottle (DO NOT AUTOCLAVE due to precipitation).

- Store at room temperature or 4°C for long-term use.

- **100 mL 50 mM Magnesium chloride solution**
  - In a clean beaker, weigh 1.017 g of $MgCl_2 \cdot 6H_2O$
  - Add 100 mL MQ water. Place a stir bar in the beaker and stir until completely dissolved.
  - Pass the solution through a 0.22 μm sterile filter into a sterile prelabelled storage bottle (DO NOT AUTOCLAVE due to precipitation).
    Store at room temperature or 4°C for long-term use.

Supplementary Figures

Figure S1. Vector maps
Figure S2. Multiplex crRNA design
Figure S3. *Agrobacterium* transformation
Figure S4. Cre-*loxP* recombination
Figure S5. PCR screening
Figure S6. K599dT WGS analysis
Figure S7. Map of pLC112K

Supplementary Tables

Table S1. List of custom oligonucleotides/primers for cloning and PCR analysis.

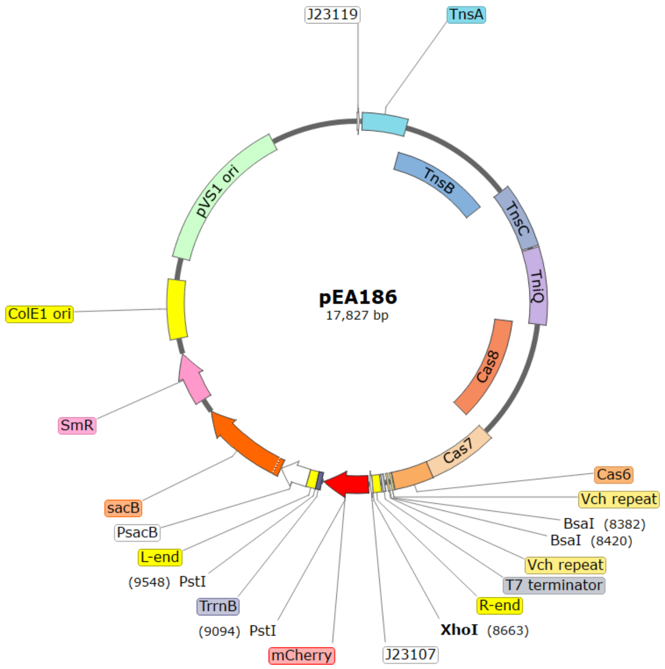

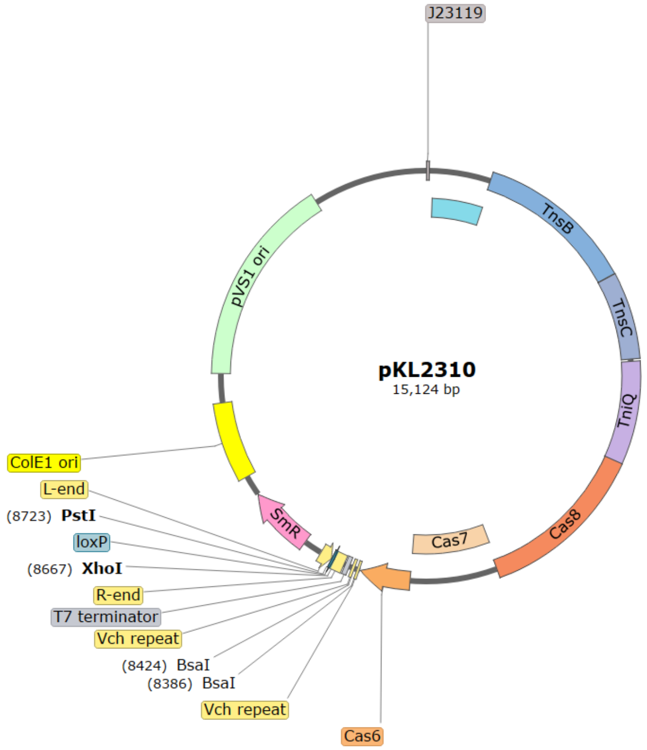

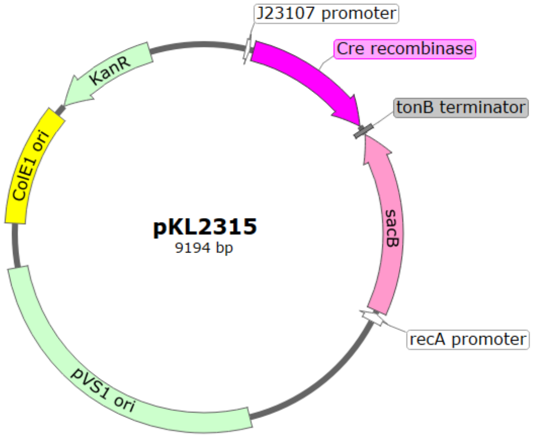

Km, Kanamycin
NA, Not Applicable
sacB, Levansucrase gene from *Bacillus subtilis*
Sp, Spectinomycin
Sm, Streptomycin
Vch, *Vibrio cholerae*

Plasmid	Addgene ID	System / Description	Antibiotic Selection	Other Selection	crRNA / Spacer	Mini-Tn Cargo	Notes
pEA186	187874	Type I-F pVSI *VchCAST*	Sp, Sm	sacB	BsaI	XhoI / PstI	Single vector for site-specific genomic integration of the INTEGRATE mini-transposon (mini-Tn) cargo in *Agrobacterium*
pKL2310	187875	Type I-F pVSI *VchCAST*	Sp, Sm	NA	BsaI	XhoI / PstI	LoxP harboring vector to mediate targeted loxP insertion for deletion assays in *Agrobacterium*
pKL2315	187876	Type I-F pVSI *VchCAST*	Km	sacB	NA	NA	Cre-expression vector to facilitate deletion between dual recombination (*lox*P) sites in *Agrobacterium*

Figure S1. INTEGRATE vectors for *Agrobacterium* genome engineering.

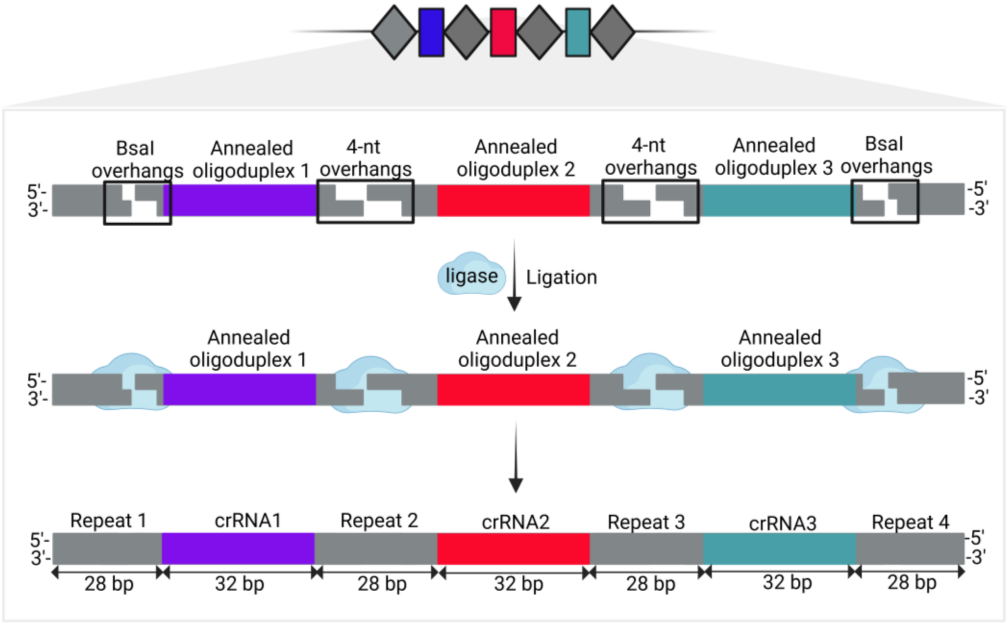

Figure S2. Multiplexed spacer ligation-mediated cloning strategy for the INTEGRATE system. The *Vibrio cholerae* INTEGRATE system features a 32-bp spacer that is complementary to the target site and is flanked by two 28-bp repeats. For the multiplexing process, each newly added spacer is also flanked by two additional spacers. A short 4-nucleotide overhang is included in the oligonucleotide design to facilitate directional ligation (refer to Table S1). The schematic representations were created using Biorender.

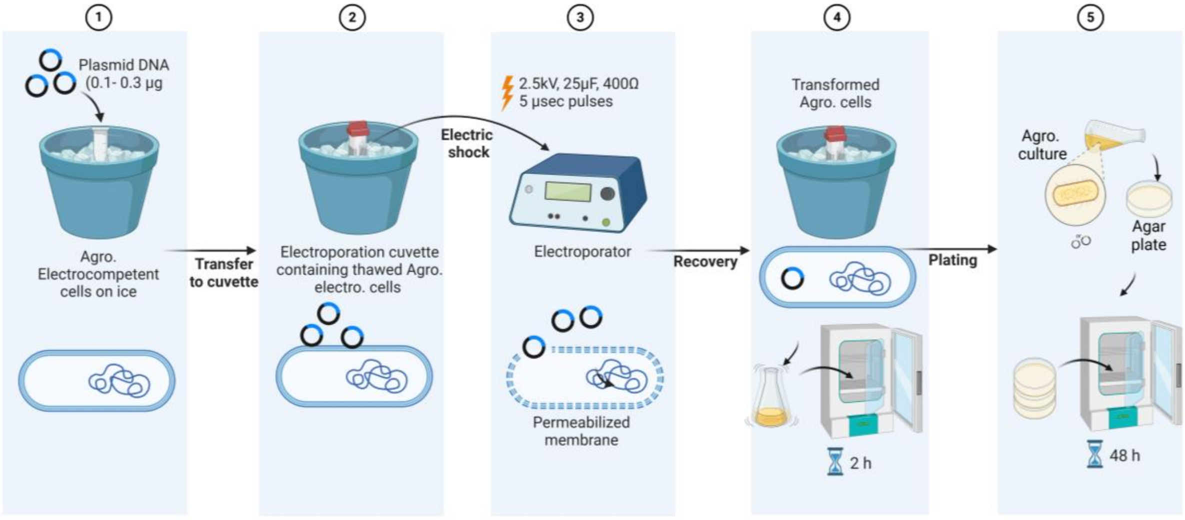

Figure S3. An overview of *Agrobacterium* transformation using electroporation. Electroporation uses an electric field to temporarily disrupt the Agrobacterial membrane, creating pores that allow plasmid DNA to enter. This method is highly efficient and is preferred for its high transformation rates in *Agrobacterium*.

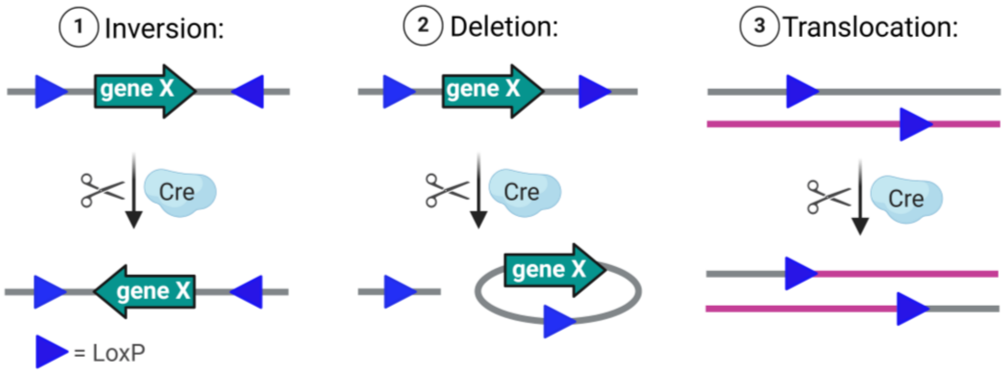

Figure S4. Potential outcomes of Cre/*lox*P recombination system. Depending on the position and orientation of the dual *lox*P sites, three different outcomes can be achieved: (1) inversion—when two *lox*P sites are located on the same DNA molecule with opposite orientations, (2) deletion—when both *lox*P sites are located on the same DNA molecule with the same orientation, and (3) translocation—when two *lox*P sites are located in different DNA molecules. The schematic representations were created using Biorender.

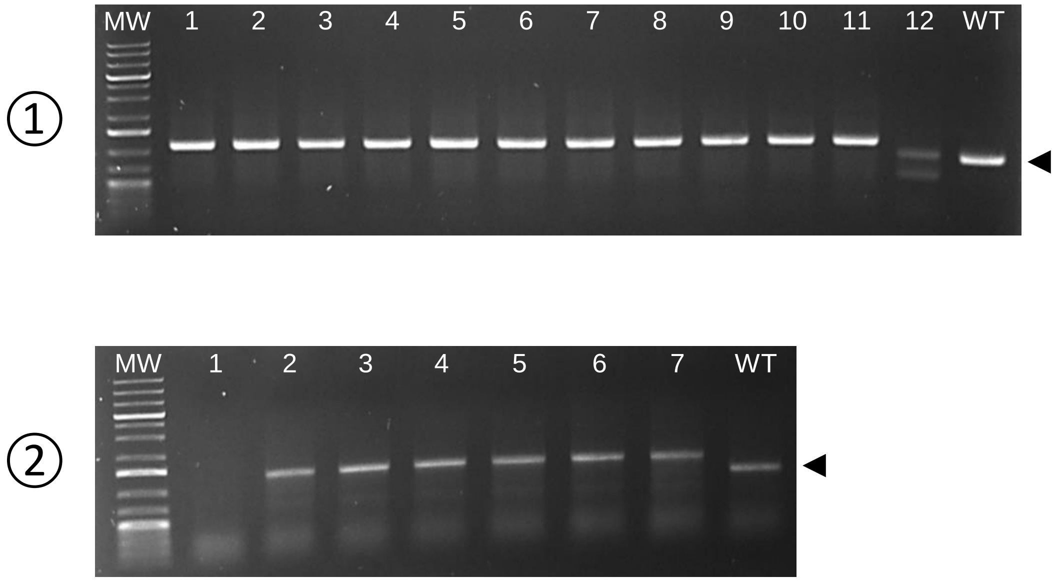

Figure S5. PCR screening of *loxP* insertion into two target sites: (1) LB target, and (2) RB target. Numbers 1-12 indicate individual colonies screened: 12 for LB target and 7 for RB target, respectively. For the LB target, all but one (#12) had targeted insertion (1,119 bp). For RT target, colonies 2-7 had targeted insertion (1,299 bp) and PCR failed for colony #1. WT, wild-type band: 810 bp and 990 bp for LB and RB targets, respectively. Black arrow heads indicates WT band without *loxP* insertion.

①

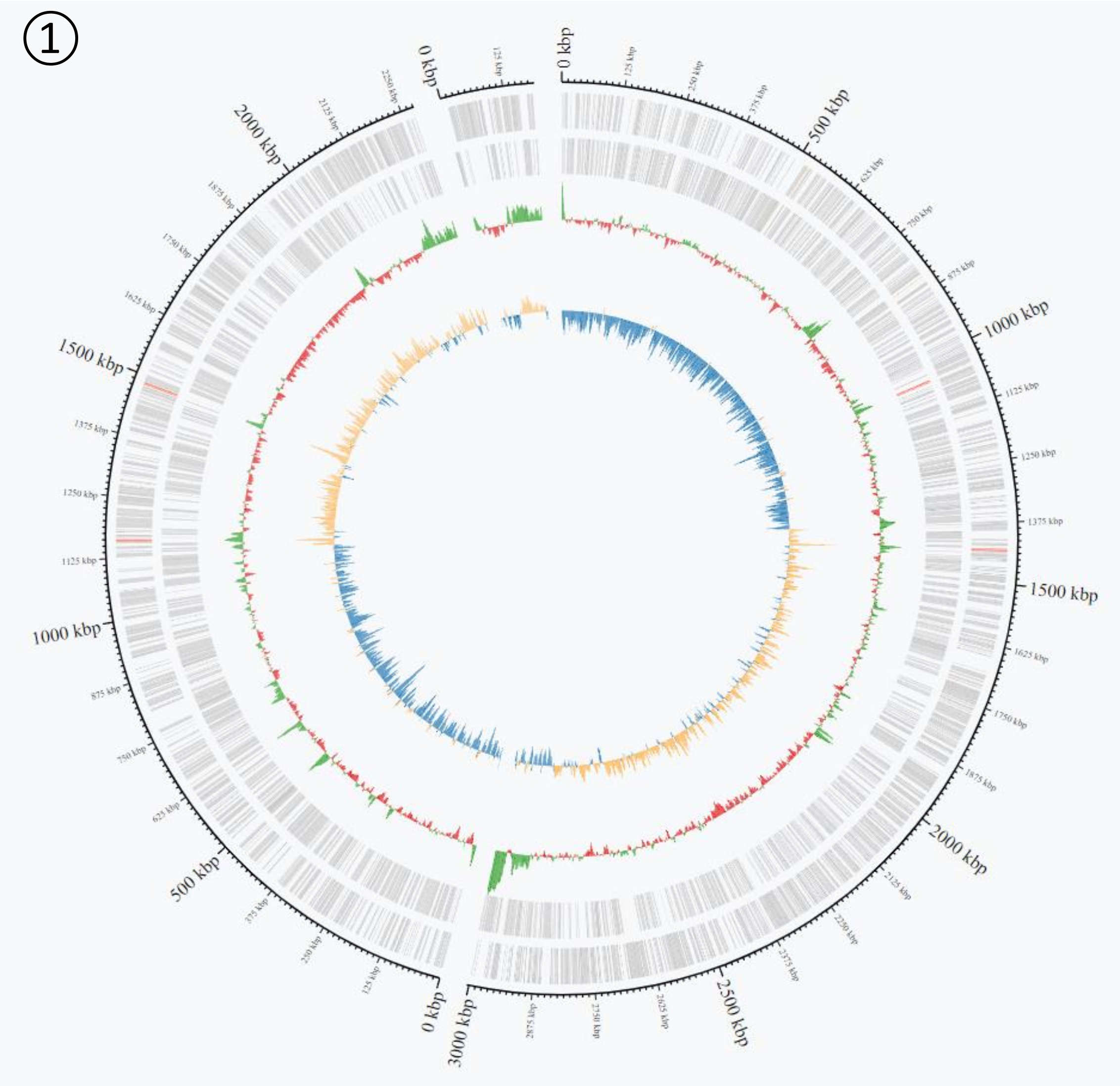

②

Strain	Chromosome 1	Chromosome 2	pRi2659	Reference
K599	3,003,350	2,276,589	202,302	Caspi et al., 2020
K599dT	3,004,037	2,276,466	187,362	Plasmidsaurus ID 3VTNQV_1

Figure S6. Whole Genome Sequencing analysis of K599dT strain. K599dT strain has a smaller pRi2659 plasmid corresponding to the precise T-DNA deletion.

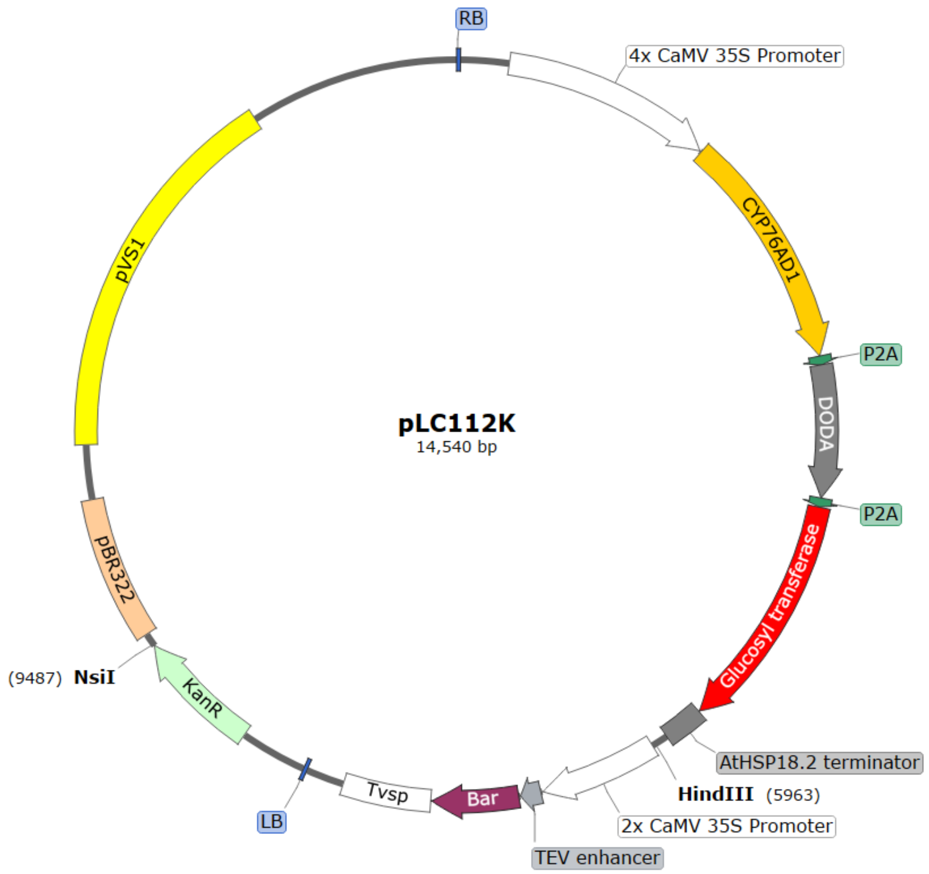

Figure S7. Map of pLC112K. RB, right border; 4x CaMV 35S promoter, Cauliflower Mosaic Virus 35S promoter with quadruple enhancers; CYP76AD1, Cytochrome P450 76AD1; P2A, self-cleaving peptide derived form the porcine teschovirus-1; DODA, L-DOPA 4,5-dioxygenase; AtHSP18.2 terminator, Arabidopsis heat shock protein gene 18.2 terminator; 2x CaMV 35S promoter, Cauliflower Mosaic Virus 35S promoter with double enhancers; TEV enhancer, Tobacco Etch Virus enhancer; bar, bialaphos resistance gene; Tvsp, soybean vegetative storage protein gene terminator; LB, left border; KanR, Kanamycin resistance gene; pBR322, origin of replication for *Escherichia coli*; pVS1, origin of replication from *Pseudomonas aeruginosa* plasmid pVS1.

Table S1. Custom oligonucleotides/primers for cloning and PCR

ID	Primer name	Sequence	Decription	Reference
1	pSL1765-seq-F1	AACGGATCCGAATTCGCTGC	Forward sequencing primer for validating cloned mini-Tn cargo.	Aliu et al., 2022
2	pSL1765-seq-R1	GTCAATTATTACCTCCACGG	Reverse sequencing primer for validating cloned mini-Tn cargo.	Aliu et al., 2023
3	pSL1765-seq-F2	ATATCAGGATGCTCTCGGAG	Forward sequencing primer for validating cloned crRNA.	Aliu et al., 2024

Single crRNA cloning custom design (5'-3'):

ID	Primer name	Sequence	Decription	Reference
4	OligoF	**ATAAC**[32-nt crRNA sequence]**G**	Forward oligo to be annealed with ID5. 5'-ATAAC and 3'-G-nt enable ligation to the BsaI-digested vector backbone, respectively.	This study
5	OligoR	**TTCAC**[reverse complement of 32-nt crRNA]**G**	5'-3' Reverse oligo to be annealed with ID4. 5'-TTCAC and 3'-G-nt enable ligation to the BsaI-digested vector backbone, respectively.	This study

Dual crRNA cloning custom design (5'-3'):

ID	Primer name	Sequence	Decription	Reference
6	OligoF1	**ATAAC**[32-nt crRNA1]GTGAACTGCCGAGTAG	Forward oligo to be annealed with OligoR1. 5'-ATAAC enable ligation to the BsaI-digested vector backbone.	This study
7	OligoR1	**CTAC**CTACTCGGCAGTTCAC[crRNA1 reverse complement]**G**	Reverse oligo to be annealed with OligoF1. 3'-G-nt enable ligation to the BsaI-digested vector backbone.	This study
8	OligoF2	**GTAG**CTGATAAC[32-nt crRNA2]**G**	Forward oligo to be annealed with OligoR2. 3'-G-nt enable ligation to the BsaI-digested vector backbone.	This study
9	OligoR2	**TTCAC**[crRNA2 reverse complement]GTTATCAG	Reverse oligo to be annealed with OligoF2. 5'-TTCAC enable ligation to the BsaI-digested vector backbone.	This study

Triple crRNA cloning custom oligo design (5'-3'):

ID	Primer name	Sequence	Decription	Reference
10	OligoF3	**ATAAC**[32-nt crRNA1]GTGAACTGCCGAGTAGGTAGCT	Forward oligo to be annealed with OligoR3. 5'-ATAAC enable ligation to the BsaI-digested vector backbone.	This study
11	OligoR3	**TATC**AGCTACCTACTCGGCAGTTCAC[crRNA1 reverse complement]**G**	Reverse oligo to be annealed with OligoF3. 5'-4-nt overhang (red) enable ligation with GATA in ID12; 3'-G-nt enable ligation to vector backbone.	This study
12	OligoF4	**GATA**AC[32-nt crRNA2]GTGAACTGCCGAGTAGGTAGC	Forward oligo to be annealed with OligoR4. 5'-4-nt overhang (red) enable ligation with TATC in ID11.	This study
13	OligoR4	**ATCA**GCTACCTACTCGGCAGTTCAC[crRNA2 reverse complement]GT	Reverse oligo to be annealed with OligoF4. 5'-4-nt overhang (purple) enable ligation with TGAT in ID14	This study
14	OligoF5	**TGAT**AAC[32-nt crRNA3]**G**	Forward oligo to be annealed with OligoR5. 5'-4-nt overhang (purple) enable ligation with ATCA in ID13; 3'-G-nt enable ligation to the BsaI-digested vector backbone.	This study
15	OligoR5	**TTCAC**[crRNA3 reverse complement]GTT	Reverse oligo to be annealed with OligoF5. 5'-TTCAC enable ligation to the BsaI-digested vector backbone.	This study

Qoudruple crRNA cloning custom oligo design (5'-3'):

ID	Primer name	Sequence	Decription	Reference
16	OligoF3	**ATAAC**[32-nt crRNA1]GTGAACTGCCGAGTAGGTAGCT	Forward oligo to be annealed with OligoR3. 5'-ATAAC enable ligation to the BsaI-digested vector backbone.	This study
17	OligoR3	**TATC**AGCTACCTACTCGGCAGTTCAC[crRNA1 reverse complement]**G**	Reverse oligo to be annealed with OligoF3. 5'-4-nt overhang (red) enable ligation with GATA in ID18; 3'-G-nt enable ligation to vector backbone.	This study
18	OligoF4	**GATA**AC[32-nt crRNA2]GTGAACTGCCGAGTAGGTAGC	Forward oligo to be annealed with OligoR4. 5'-4-nt overhang (red) enable ligation with TATC in ID17.	This study
19	OligoR4	**ATCA**GCTACCTACTCGGCAGTTCAC[crRNA2 reverse complement]GT	Reverse oligo to be annealed with OligoF4. 5'-4-nt overhang (purple) enable ligation with TGAT in ID20	This study
20	OligoF6	**TGAT**AAC[32-nt crRNA3]GTGAACTGCCGAGTAGGTAG	Forward oligo to be annealed with OligoR6. 5'-4-nt overhang (purple) enable ligation with ATCA in ID19.	This study
21	OligoR6	**TCAG**CTACCTACTCGGCAGTTCAC[crRNA3 reverse complement]GTT	Reverse oligo to be annealed with OligoR6. 5'-4-nt overhang (green) enable ligation with CTGA in ID22	This study
22	OligoF7	**CTGA**TAAC[32-nt crRNA4]**G**	Forward oligo to be annealed with OligoR7. 5'-4-nt overhang (purple) enable ligation with TCAG in ID21; 3'-G-nt enable ligation to the BsaI-digested vector backbone.	This study
23	OligoR7	**TTCAC**[crRNA4 reverse complement]GTTA	Reverse oligo to be annealed with OligoF7. 5'-TTCAC enable ligation to the BsaI-digested vector backbone.	This study

XhoI/PstI-mediated directional cloning custom oligo design (5'-3'):

ID	Primer name	Sequence	Decription	Reference
24	OligoF10-XhoI	**CCGCTCGAG[**Forward primer sequence here]	Forward custom design primer combined with oligoR10-PstI for amplifying a desired sequence. For efficient end cleavage of a XhoI recognition site (purple), NEB recommends including at least 3 nts (red) at the 5' end of your primer as shown.	This study
25	OligoR10-PstI	AACTGCAG[Reverse primer sequence here]	Reverse custom design primer combined with oligoF10-XhoI for amplifying a desired sequence. For efficient end cleavage of a PstI recognition site (purple), NEB recommends including at least 2 nts (red) at the 5' end of your primer as shown.	This study

*nt, nucleotide; crRNA, guide RNA sequence; 5'-3', reading frame.
